# Supplementary material for: Exploring the evidence of direct threats to cetaceans from maritime vessels: A systematic map
Source: PLoS One. 2026 May 7;21(5):e0348502. doi: 10.1371/journal.pone.0348502 (PMC13152187; doi:10.1371/journal.pone.0348502)
Supplement: S1 Appendix — (DOCX) [file pone.0348502.s001.docx]

**S1 Table.** Details of the search databases, strings and dates used to perform a systematic map exercise on the primary impacts of maritime vessels on marine cetaceans. All strings followed this structure: [cetacean-related terms] AND [stressor-related terms].

| **Search Engine** | **Search Date** | **Search String** | **Number of Results** |
| --- | --- | --- | --- |
| Scopus  no time limit | 31/01/2023 | [(cetacea* OR dolphin* OR whale* OR porpoise* OR orca OR vaquita OR beluga OR narwhal AND NOT "river dolphin*") OR ("Balaena mysticetus" OR "Eubalaena glacialis" OR "Eubalaena japonica" OR "Eubalaena australis" OR "Balaenoptera musculus" OR "Balaenoptera brydei" OR "Balaenoptera edeni" OR "Balaenoptera acutorostrata" OR "Balaenoptera ricei" OR "Balaenoptera physalus" OR "Balaenoptera omurai" OR "Balaenoptera borealis" OR "Balaenoptera bonaerensi"s OR "Megaptera novaeangliae" OR "Eschrichtius robustu"s OR "Caperea marginata" OR "Cephalorhynchus eutropia" OR "Cephalorhynchus commersonii" OR "Cephalorhynchus heavisidii" OR "Cephalorhynchus hectori" OR "Delphinus delphis" OR "Feresa attenuata" OR "Globicephala melas" OR "Globicephala macrorhynchus" OR "Grampus griseus" OR "Lagenodelphis hosei" OR "Lagenorhynchus albirostris" OR "Lagenorhynchus acutus" OR "Lagenorhynchus obscurus" OR "Lagenorhynchus cruciger" OR "Lagenorhynchus obliquidens" OR "Lagenorhynchus australis" OR "Lissodelphis borealis" OR "Lissodelphis peronii" OR "Orcaella heinsohni" OR "Orcaella brevirostris" OR "Orcinus orca" OR "Peponocephala electra" OR "Pseudorca crassidens" OR "Sousa teuszii" OR "Sousa sahulensis" OR "Sousa plumbea" OR "Sousa chinensis" OR "Sotalia guianensis" OR "Stenella frontalis" OR "Stenella clymene" OR "Stenella attenuata" OR "Stenella longirostris" OR "Stenella coeruleoalba" OR "Steno bredanensis" OR "Tursiops truncatus" OR "Tursiops aduncus" OR "Delphinapterus leucas" OR "Monodon monoceros" OR "Neophocaena phocaenoides" OR "Neophocaena asiaeorientalis" OR "Phocaena spinipinnis" OR "Phocaena phocaena" OR "Phocaena dioptrica" OR "Phocaena sinus" OR "Phocoenoides dalli" OR "Physeter macrocephalus" OR "Kogia sima" OR "Kogia breviceps" OR "Berardius arnuxii" OR "Berardius bairdii" OR "Berardius minimus" OR "Tasmacetus shepherdi" OR "Ziphius cavirostris" OR "Hyperoodon ampullatus" OR "Hyperoodon planifrons" OR "Indopacetus pacificus" OR "Mesoplodon bowdoini" OR "Mesoplodon densirostris" OR "Mesoplodon hotaula" OR "Mesoplodon ginkgodens" OR "Mesoplodon grayi" OR "Mesoplodon hectori" OR "Mesoplodon carlhubbsi" OR "Mesoplodon perrini" OR "Mesoplodon peruvianus" OR "Mesoplodon eueu" OR "Mesoplodon bidens" OR "Mesoplodon traversii" OR "Mesoplodon bahamondi" OR "Mesoplodon stejnegeri" OR "Mesoplodon layardii" OR "Mesoplodon mirus") OR ("B. mysticetus" OR "E. glacialis" OR "E. japonica" OR "E. australis" OR "B. musculus" OR "B. brydei" OR "B. edeni" OR "B. acutorostrata" OR "B. ricei" OR "B. physalus" OR "B. omurai" OR "B. borealis" OR "B. bonaerensis" OR "M. novaeangliae" OR "E. robustus" OR "C. marginata" OR "C. eutropia" OR "C. commersonii" OR "C. heavisidii" OR "C. hectori" OR "D. delphis" OR "F. attenuata" OR "G. melas" OR "G. macrorhynchus" OR "G. griseus" OR "L. hosei" OR "L. albirostris" OR "L. acutus" OR "L. obscurus" OR "L. cruciger" OR "L. obliquidens" OR "L. australis" OR "L. borealis" OR "L. peronii" OR "O. heinsohni" OR "O. brevirostris" OR "O. orca" OR "P. electra" OR "P. crassidens" OR "S. teuszii" OR "S. sahulensis" OR "S. plumbea" OR "S. chinensis" OR "S. guianensis" OR "S. frontalis" OR "S. clymene" OR "S. attenuata" OR "S. longirostris" OR "S. coeruleoalba" OR "S. bredanensis" OR "T. truncatus" OR "T. aduncus" OR "D. leucas" OR "M. monoceros" OR "N. phocaenoides" OR "N. asiaeorientalis" OR "P. spinipinnis" OR "P. phocaena" OR "P. dioptrica" OR "P. sinus" OR "P. dalli" OR "P. macrocephalus" OR "K. sima" OR "K. breviceps" OR "B. arnuxii" OR "B. bairdii" OR "B. minimus" OR "T. shepherdi" OR "Z. cavirostris" OR "H. ampullatus" OR "H. planifrons" OR "I. pacificus" OR "M. bowdoini" OR "M. densirostris" OR "M. hotaula" OR "M. ginkgodens" OR "M. grayi" OR "M. hectori" OR "M. carlhubbsi" OR "M. perrini" OR "M. peruvianus" OR "M. eueu" OR "M. bidens" OR "M. traversii" OR "M. bahamondi" OR "M. stejnegeri" OR "M. layardii" OR "M. mirus")] AND [boat* OR vessel* OR ship* OR craft OR “whale watching” OR ferr* OR cruise* OR liner OR cargo OR tanker OR freighter OR container OR icebreaker OR tugboat OR tug OR trawl* OR yacht OR jetski OR {jet ski} OR {jet drive} OR sail OR motorboat OR speedboat OR commercial OR recreational OR traffic OR "coast guard" OR hovercraft OR hydrofoil OR catamaran OR submarine OR military OR regatta OR dredg* OR port OR harbour OR harbor] | 6951 |
| Web of Science – All | 31/01/2023 | [(cetacea* OR dolphin* OR whale* OR porpoise* OR orca OR vaquita OR beluga OR narwhal NOT "river dolphin*") OR ("Balaena mysticetus" OR "Eubalaena glacialis" OR "Eubalaena japonica" OR "Eubalaena australis" OR "Balaenoptera musculus" OR "Balaenoptera brydei" OR "Balaenoptera edeni" OR "Balaenoptera acutorostrata" OR "Balaenoptera ricei" OR "Balaenoptera physalus" OR "Balaenoptera omurai" OR "Balaenoptera borealis" OR "Balaenoptera bonaerensi"s OR "Megaptera novaeangliae" OR "Eschrichtius robustu"s OR "Caperea marginata" OR "Cephalorhynchus eutropia" OR "Cephalorhynchus | 5374 |

**S1 Table continued.**

| **Search Engine** | **Search Date** | **Search String** | **Number of Results** |
| --- | --- | --- | --- |
| Web of Science – All Collections  no time limit | 31/01/2023 | [(cetacea* OR dolphin* OR whale* OR porpoise* OR orca OR vaquita OR beluga OR narwhal NOT "river dolphin*") OR ("Balaena mysticetus" OR "Eubalaena glacialis" OR "Eubalaena japonica" OR "Eubalaena australis" OR "Balaenoptera musculus" OR "Balaenoptera brydei" OR "Balaenoptera edeni" OR "Balaenoptera acutorostrata" OR "Balaenoptera ricei" OR "Balaenoptera physalus" OR "Balaenoptera omurai" OR "Balaenoptera borealis" OR "Balaenoptera bonaerensi"s OR "Megaptera novaeangliae" OR "Eschrichtius robustu"s OR "Caperea marginata" OR "Cephalorhynchus eutropia" OR "Cephalorhynchus commersonii" OR "Cephalorhynchus heavisidii" OR "Cephalorhynchus hectori" OR "Delphinus delphis" OR "Feresa attenuata" OR "Globicephala melas" OR "Globicephala macrorhynchus" OR "Grampus griseus" OR "Lagenodelphis hosei" OR "Lagenorhynchus albirostris" OR "Lagenorhynchus acutus" OR "Lagenorhynchus obscurus" OR "Lagenorhynchus cruciger" OR "Lagenorhynchus obliquidens" OR "Lagenorhynchus australis" OR "Lissodelphis borealis" OR "Lissodelphis peronii" OR "Orcaella heinsohni" OR "Orcaella brevirostris" OR "Orcinus orca" OR "Peponocephala electra" OR "Pseudorca crassidens" OR "Sousa teuszii" OR "Sousa sahulensis" OR "Sousa plumbea" OR "Sousa chinensis" OR "Sotalia guianensis" OR "Stenella frontalis" OR "Stenella clymene" OR "Stenella attenuata" OR "Stenella longirostris" OR "Stenella coeruleoalba" OR "Steno bredanensis" OR "Tursiops truncatus" OR "Tursiops aduncus" OR "Delphinapterus leucas" OR "Monodon monoceros" OR "Neophocaena phocaenoides" OR "Neophocaena asiaeorientalis" OR "Phocaena spinipinnis" OR "Phocaena phocaena" OR "Phocaena dioptrica" OR "Phocaena sinus" OR "Phocoenoides dalli" OR "Physeter macrocephalus" OR "Kogia sima" OR "Kogia breviceps" OR "Berardius arnuxii" OR "Berardius bairdii" OR "Berardius minimus" OR "Tasmacetus shepherdi" OR "Ziphius cavirostris" OR "Hyperoodon ampullatus" OR "Hyperoodon planifrons" OR "Indopacetus pacificus" OR "Mesoplodon bowdoini" OR "Mesoplodon densirostris" OR "Mesoplodon hotaula" OR "Mesoplodon ginkgodens" OR "Mesoplodon grayi" OR "Mesoplodon hectori" OR "Mesoplodon carlhubbsi" OR "Mesoplodon perrini" OR "Mesoplodon peruvianus" OR "Mesoplodon eueu" OR "Mesoplodon bidens" OR "Mesoplodon traversii" OR "Mesoplodon bahamondi" OR "Mesoplodon stejnegeri" OR "Mesoplodon layardii" OR "Mesoplodon mirus") OR ("B. mysticetus" OR "E. glacialis" OR "E. japonica" OR "E. australis" OR "B. musculus" OR "B. brydei" OR "B. edeni" OR "B. acutorostrata" OR "B. ricei" OR "B. physalus" OR "B. omurai" OR "B. borealis" OR "B. bonaerensis" OR "M. novaeangliae" OR "E. robustus" OR "C. marginata" OR "C. eutropia" OR "C. commersonii" OR "C. heavisidii" OR "C. hectori" OR "D. delphis" OR "F. attenuata" OR "G. melas" OR "G. macrorhynchus" OR "G. griseus" OR "L. hosei" OR "L. albirostris" OR "L. acutus" OR "L. obscurus" OR "L. cruciger" OR "L. obliquidens" OR "L. australis" OR "L. borealis" OR "L. peronii" OR "O. heinsohni" OR "O. brevirostris" OR "O. orca" OR "P. electra" OR "P. crassidens" OR "S. teuszii" OR "S. sahulensis" OR "S. plumbea" OR "S. chinensis" OR "S. guianensis" OR "S. frontalis" OR "S. clymene" OR "S. attenuata" OR "S. longirostris" OR "S. coeruleoalba" OR "S. bredanensis" OR "T. truncatus" OR "T. aduncus" OR "D. leucas" OR "M. monoceros" OR "N. phocaenoides" OR "N. asiaeorientalis" OR "P. spinipinnis" OR "P. phocaena" OR "P. dioptrica" OR "P. sinus" OR "P. dalli" OR "P. macrocephalus" OR "K. sima" OR "K. breviceps" OR "B. arnuxii" OR "B. bairdii" OR "B. minimus" OR "T. shepherdi" OR "Z. cavirostris" OR "H. ampullatus" OR "H. planifrons" OR "I. pacificus" OR "M. bowdoini" OR "M. densirostris" OR "M. hotaula" OR "M. ginkgodens" OR "M. grayi" OR "M. hectori" OR "M. carlhubbsi" OR "M. perrini" OR "M. peruvianus" OR "M. eueu" OR "M. bidens" OR "M. traversii" OR "M. bahamondi" OR "M. stejnegeri" OR "M. layardii" OR "M. mirus")] AND [boat* OR vessel* OR ship* OR craft OR “whale watching” OR ferr* OR cruise* OR liner OR cargo OR tanker OR freighter OR container OR icebreaker OR tugboat OR tug OR trawl* OR yacht OR jetski OR {jet ski} OR {jet drive} OR sail OR motorboat OR speedboat OR commercial OR recreational OR traffic OR "coast guard" OR hovercraft OR hydrofoil OR catamaran OR submarine OR military OR regatta OR dredg* OR port OR harbour OR harbor] | 5374 |
| ProQuest | 31/01/2023 | [(cetacea* OR dolphin* OR whale* OR porpoise* OR orca OR vaquita OR beluga OR narwhal NOT "river dolphin*") OR ("Balaena mysticetus" OR "Eubalaena glacialis" OR "Eubalaena japonica" OR "Eubalaena australis" OR "Balaenoptera musculus" OR "Balaenoptera brydei" OR "Balaenoptera edeni" OR "Balaenoptera acutorostrata" OR "Balaenoptera ricei" OR "Balaenoptera physalus" OR "Balaenoptera omurai" OR "Balaenoptera borealis" OR "Balaenoptera bonaerensi"s OR "Megaptera novaeangliae" OR "Eschrichtius robustu"s OR "Caperea marginata" OR "Cephalorhynchus eutropia" OR "Cephalorhynchus commersonii" OR "Cephalorhynchus heavisidii" OR "Cephalorhynchus hectori" OR "Delphinus delphis" OR "Feresa attenuata" OR "Globicephala melas" OR "Globicephala macrorhynchus" OR "Grampus griseus" OR "Lagenodelphis hosei" OR "Lagenorhynchus albirostris" OR "Lagenorhynchus acutus" OR "Lagenorhynchus obscurus" OR "Lagenorhynchus cruciger" OR "Lagenorhynchus obliquidens" OR "Lagenorhynchus australis" OR "Lissodelphis borealis" OR "Lissodelphis peronii" OR "Orcaella heinsohni" OR "Orcaella brevirostris" OR "Orcinus orca" OR "Peponocephala electra" OR "Pseudorca crassidens" OR "Sousa teuszii" OR "Sousa sahulensis" OR "Sousa plumbea" OR "Sousa chinensis" OR "Sotalia guianensis" OR "Stenella frontalis" OR "Stenella clymene" OR "Stenella attenuata" OR "Stenella longirostris" OR "Stenella coeruleoalba" OR "Steno bredanensis" OR "Tursiops truncatus" OR "Tursiops aduncus" OR "Delphinapterus leucas" OR "Monodon monoceros" OR "Neophocaena phocaenoides" OR "Neophocaena asiaeorientalis" OR "Phocaena spinipinnis" OR "Phocaena phocaena" OR "Phocaena dioptrica" OR "Phocaena sinus" OR "Phocoenoides dalli" OR "Physeter macrocephalus" OR "Kogia sima" OR "Kogia breviceps" OR "Berardius arnuxii" OR "Berardius bairdii" OR "Berardius minimus" OR "Tasmacetus shepherdi" OR "Ziphius cavirostris" OR "Hyperoodon ampullatus" OR "Hyperoodon planifrons" OR "Indopacetus pacificus" OR "Mesoplodon bowdoini" OR "Mesoplodon densirostris" OR "Mesoplodon hotaula" OR "Mesoplodon ginkgodens" OR "Mesoplodon grayi" OR "Mesoplodon hectori" OR "Mesoplodon carlhubbsi" OR "Mesoplodon perrini" OR "Mesoplodon peruvianus" OR "Mesoplodon eueu" OR "Mesoplodon bidens" OR "Mesoplodon traversii" OR "Mesoplodon bahamondi" OR "Mesoplodon stejnegeri" OR "Mesoplodon layardii" OR "Mesoplodon mirus") OR ("B. mysticetus" OR "E. glacialis" OR "E. japonica" OR "E. australis" OR "B. musculus" OR "B. brydei" OR "B. edeni" OR "B. acutorostrata" OR "B. ricei" OR "B. physalus" OR "B. omurai" OR "B. borealis" OR "B. bonaerensis" OR "M. novaeangliae" OR "E. robustus" OR "C. marginata" OR "C. eutropia" OR "C. commersonii" OR "C. heavisidii" OR "C. hectori" OR "D. delphis" OR "F. attenuata" OR "G. melas" OR "G. macrorhynchus" OR "G. griseus" OR "L. hosei" OR "L. albirostris" OR "L. acutus" OR "L. obscurus" OR "L. cruciger" OR "L. obliquidens" OR "L. australis" OR "L. borealis" OR "L. peronii" OR "O. heinsohni" OR "O. brevirostris" OR "O. orca" OR "P. electra" OR "P. crassidens" OR "S. teuszii" OR "S. sahulensis" OR "S. plumbea" OR "S. chinensis" OR "S. guianensis" OR "S. frontalis" OR "S. clymene" OR "S. attenuata" OR "S. longirostris" OR "S. coeruleoalba" OR "S. bredanensis" OR "T. truncatus" OR "T. aduncus" OR "D. leucas" OR "M. monoceros" OR "N. phocaenoides" OR "N. asiaeorientalis" OR "P. spinipinnis" OR "P. phocaena" OR "P. dioptrica" OR "P. sinus" OR "P. dalli" OR "P. macrocephalus" OR "K. sima" OR "K. breviceps" OR "B. arnuxii" OR "B. bairdii" OR "B. minimus" OR "T. shepherdi" OR "Z. cavirostris" OR "H. ampullatus" OR "H. planifrons" OR "I. pacificus" OR "M. bowdoini" OR "M. densirostris" OR "M. hotaula" OR "M. ginkgodens" OR "M. grayi" OR "M. hectori" OR "M. carlhubbsi" OR "M. perrini" OR "M. peruvianus" OR "M. eueu" OR "M. bidens" OR "M. traversii" OR "M. bahamondi" OR "M. stejnegeri" OR "M. layardii" OR "M. mirus")] AND [boat* OR vessel* OR ship* OR craft OR “whale watching” OR ferr* OR cruise* OR liner OR cargo OR tanker OR freighter OR container OR icebreaker OR tugboat OR tug OR trawl* OR yacht OR jetski OR exact(jet ski) OR exact(jet drive) OR sail OR motorboat OR speedboat OR commercial OR recreational OR traffic OR "coast guard" OR hovercraft OR hydrofoil OR catamaran OR submarine OR military OR regatta OR dredg* OR port OR harbour OR harbor] | 16127 |

**S2 Table.** List of cetacean species for which records were found and included in this systematic map. The table reports the common and scientific names, families and the species group they were assigned to for this review.

| **Common Name** | **Species Group** | **Family** | **Scientific Name** |
| --- | --- | --- | --- |
| Bowhead Whale | Baleen Whales | Balaenidae | Balaena mysticetus |
| North Atlantic Right Whale | Baleen Whales | Balaenidae | Eubalaena glacialis |
| North Pacific Right Whale | Baleen Whales | Balaenidae | Eubalaena japonica |
| Southern Right Whale | Baleen Whales | Balaenidae | Eubalaena australis |
| Blue Whale | Baleen Whales | Balaenopteridae | Balaenoptera musculus |
| Bryde's Whale | Baleen Whales | Balaenopteridae | Balaenoptera brydei |
| Eden's Whale | Baleen Whales | Balaenopteridae | Balaenoptera edeni |
| Minke Whale | Baleen Whales | Balaenopteridae | Balaenoptera acutorostrata |
| Rice Whale | Baleen Whales | Balaenopteridae | Balaenoptera ricei |
| Fin Whale | Baleen Whales | Balaenopteridae | Balaenoptera physalus |
| Omura's Whale | Baleen Whales | Balaenopteridae | Balaenoptera omurai |
| Sei Whale | Baleen Whales | Balaenopteridae | Balaenoptera borealis |
| Antarctic Minke Whale | Baleen Whales | Balaenopteridae | Balaenoptera bonaerensis |
| Humpback Whale | Baleen Whales | Balaenopteridae | Megaptera novaeangliae |
| Gray Whale | Baleen Whales | Eschrichtiidae | Eschrichtius robustus |
| Pygmy Right Whale | Baleen Whales | Neobalaenidae | Caperea marginata |
| Chilean Dolphin | Delphinids | Delphinidae | Cephalorhynchus eutropia |
| Commerson's Dolphin | Delphinids | Delphinidae | Cephalorhynchus commersonii |
| Heaviside's Dolphin | Delphinids | Delphinidae | Cephalorhynchus heavisidii |
| Hector's Dolphin | Delphinids | Delphinidae | Cephalorhynchus hectori |
| Common Dolphin | Delphinids | Delphinidae | Delphinus delphis |
| Pygmy Killer Whale | Delphinids | Delphinidae | Feresa attenuata |
| Long-finned Pilot Whale | Delphinids | Delphinidae | Globicephala melas |
| Short-finned Pilot Whale | Delphinids | Delphinidae | Globicephala macrorhynchus |
| Risso's Dolphin | Delphinids | Delphinidae | Grampus griseus |
| Fraser's Dolphin | Delphinids | Delphinidae | Lagenodelphis hosei |
| White-beaked Dolphin | Delphinids | Delphinidae | Lagenorhynchus albirostris |
| Atlantic White-sided Dolphin | Delphinids | Delphinidae | Lagenorhynchus acutus |
| Dusky Dolphin | Delphinids | Delphinidae | Lagenorhynchus obscurus |
| Hourglass Dolphin | Delphinids | Delphinidae | Lagenorhynchus cruciger |
| Pacific White-sided Dolphin | Delphinids | Delphinidae | Lagenorhynchus obliquidens |
| Peale's Dolphin | Delphinids | Delphinidae | Lagenorhynchus australis |
| Northern Right Whale Dolphin | Delphinids | Delphinidae | Lissodelphis borealis |
| Southern Right Whale Dolphin | Delphinids | Delphinidae | Lissodelphis peronii |
| Australian Snubfin Dolphin | Delphinids | Delphinidae | Orcaella heinsohni |
| Irrawaddy Dolphin | Delphinids | Delphinidae | Orcaella brevirostris |
| Killer Whale | Delphinids | Delphinidae | Orcinus orca |
| Melon-headed Whale | Delphinids | Delphinidae | Peponocephala electra |
| False Killer Whale | Delphinids | Delphinidae | Pseudorca crassidens |
| Atlantic Humpback Dolphin | Delphinids | Delphinidae | Sousa teuszii |
| Australian Humpback Dolphin | Delphinids | Delphinidae | Sousa sahulensis |
| Indian Ocean Humpback Dolphin | Delphinids | Delphinidae | Sousa plumbea |
| Indo-Pacific Humpback Dolphin | Delphinids | Delphinidae | Sousa chinensis |
| Guiana Dolphin | Delphinids | Delphinidae | Sotalia guianensis |
| Atlantic Spotted Dolphin | Delphinids | Delphinidae | Stenella frontalis |
| Clymene Dolphin | Delphinids | Delphinidae | Stenella clymene |
| Pantropical Spotted Dolphin | Delphinids | Delphinidae | Stenella attenuata |
| Spinner Dolphin | Delphinids | Delphinidae | Stenella longirostris |
| Striped Dolphin | Delphinids | Delphinidae | Stenella coeruleoalba |
| Rough-toothed Dolphin | Delphinids | Delphinidae | Steno bredanensis |
| Atlantic Bottlenose Dolphin | Delphinids | Delphinidae | Tursiops truncatus |
| Indo-Pacific Bottlenose Dolphin | Delphinids | Delphinidae | Tursiops aduncus |
| Franciscana Dolphin | Delphinids | Pontoporiidae | Pontoporia blainvillei |
| Beluga | Delphinids | Monodontidae | Delphinapterus leucas |
| Narwhal | Delphinids | Monodontidae | Monodon monoceros |
| Indo-Pacific Finless Porpoise | Porpoises | Phocoenidae | Neophocaena phocaenoides |
| Narrow-ridged Finless Porpoise | Porpoises | Phocoenidae | Neophocaena asiaeorientalis |

**S2 Table. continued**

| **Common Name** | **Species Group** | **Family** | **Scientific Name** |
| --- | --- | --- | --- |
| Burmeister's Porpoise | Porpoises | Phocoenidae | Phocaena spinipinnis |
| Harbour Porpoise | Porpoises | Phocoenidae | Phocaena phocaena |
| Spectacled Porpoise | Porpoises | Phocoenidae | Phocaena dioptrica |
| Vaquita | Porpoises | Phocoenidae | Phocaena sinus |
| Dall's Porpoise | Porpoises | Phocoenidae | Phocoenoides dalli |
| Sperm Whale | Sperm Whales | Physeteridae | Physeter macrocephalus |
| Dwarf Sperm Whale | Sperm Whales | Kogiidae | Kogia sima |
| Pygmy Sperm Whale | Sperm Whales | Kogiidae | Kogia breviceps |
| Arnoux Beaked Whale | Beaked Whales | Ziphiidae | Berardius arnuxii |
| Baird's Beaked Whale | Beaked Whales | Ziphiidae | Berardius bairdii |
| Sato's Beaked Whale | Beaked Whales | Ziphiidae | Berardius minimus |
| Shepherd's Beaked Whale | Beaked Whales | Ziphiidae | Tasmacetus shepherdi |
| Goose-beaked Whale | Beaked Whales | Ziphiidae | Ziphius cavirostris |
| Northern Bottlenose Whale | Beaked Whales | Ziphiidae | Hyperoodon ampullatus |
| Southern Bottlenose Whale | Beaked Whales | Ziphiidae | Hyperoodon planifrons |
| Tropical Bottlenose Whale | Beaked Whales | Ziphiidae | Indopacetus pacificus |
| Andrew's Beaked Whale | Beaked Whales | Ziphiidae | Mesoplodon bowdoini |
| Blainville's Beaked Whale | Beaked Whales | Ziphiidae | Mesoplodon densirostris |
| Deraniyagala's Beaked Whale | Beaked Whales | Ziphiidae | Mesoplodon hotaula |
| Ginkgo-toothed Beaked Whale | Beaked Whales | Ziphiidae | Mesoplodon ginkgodens |
| Gray's Beaked Whale | Beaked Whales | Ziphiidae | Mesoplodon grayi |
| Hector's Beaked Whale | Beaked Whales | Ziphiidae | Mesoplodon hectori |
| Hubb's Beaked Whale | Beaked Whales | Ziphiidae | Mesoplodon carlhubbsi |
| Perrin's Beaked Whale | Beaked Whales | Ziphiidae | Mesoplodon perrini |
| Pygmy Beaked Whale | Beaked Whales | Ziphiidae | Mesoplodon peruvianus |
| Ramari's Beaked Whale | Beaked Whales | Ziphiidae | Mesoplodon eueu |
| Sowerby's Beaked Whale | Beaked Whales | Ziphiidae | Mesoplodon bidens |
| Spade-toothed Beaked Whale | Beaked Whales | Ziphiidae | Mesoplodon traversii |
| Stejneger's Beaked Whale | Beaked Whales | Ziphiidae | Mesoplodon stejnegeri |
| Strap-toothed Beaked Whale | Beaked Whales | Ziphiidae | Mesoplodon layardii |
| True's Beaked Whale | Beaked Whales | Ziphiidae | Mesoplodon mirus |
| Gervais' Beaked whale | Beaked Whales | Ziphiidae | Mesoplodon europaeus |

**S3 Table.** Number of empirical, predictive and total amount of records found for this systematic map for each searched species.


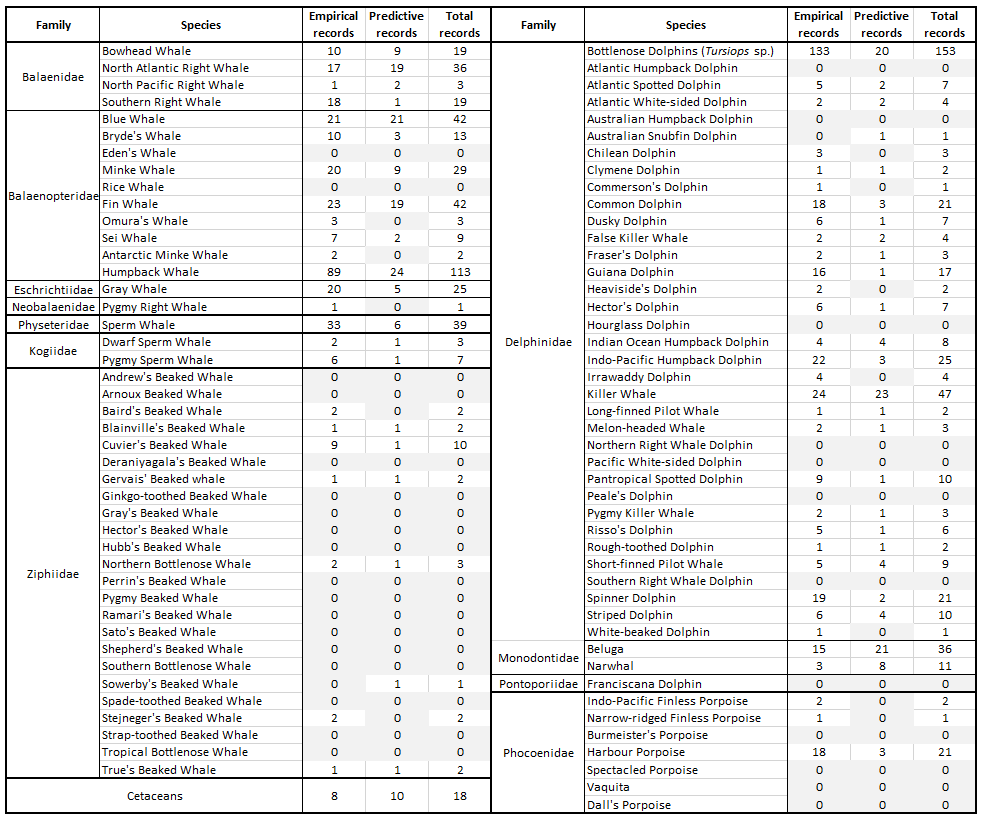


**S4 Table.** Number of empirical records found for each species group for the different time periods (A) and marine areas (B), and average number of species studied per year during the different time periods (C).


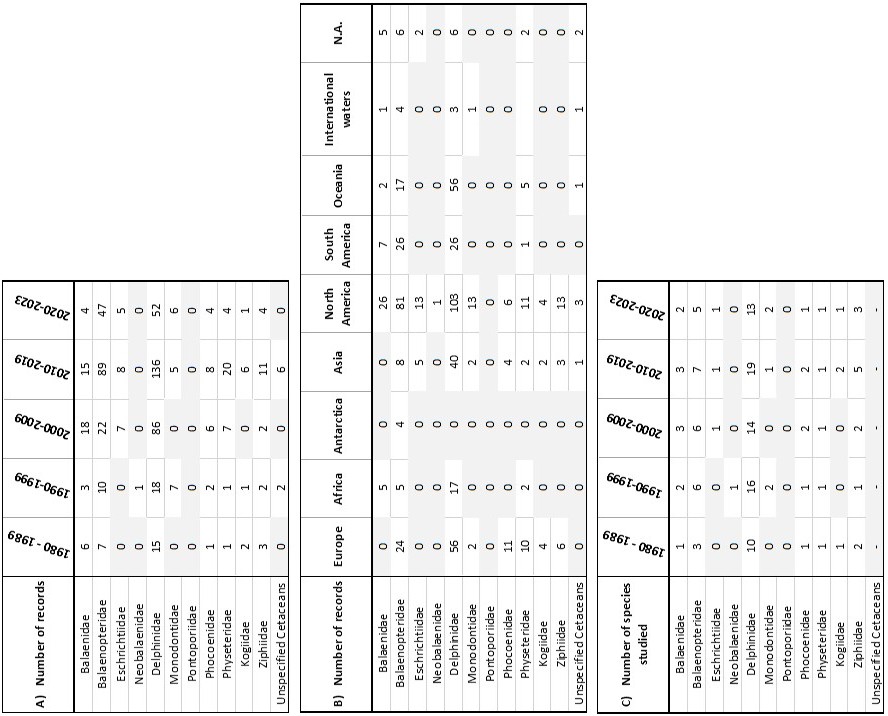


**S5 Table.** Number of empirical records found for each vessel type for the different time periods (A) and marine areas (B).


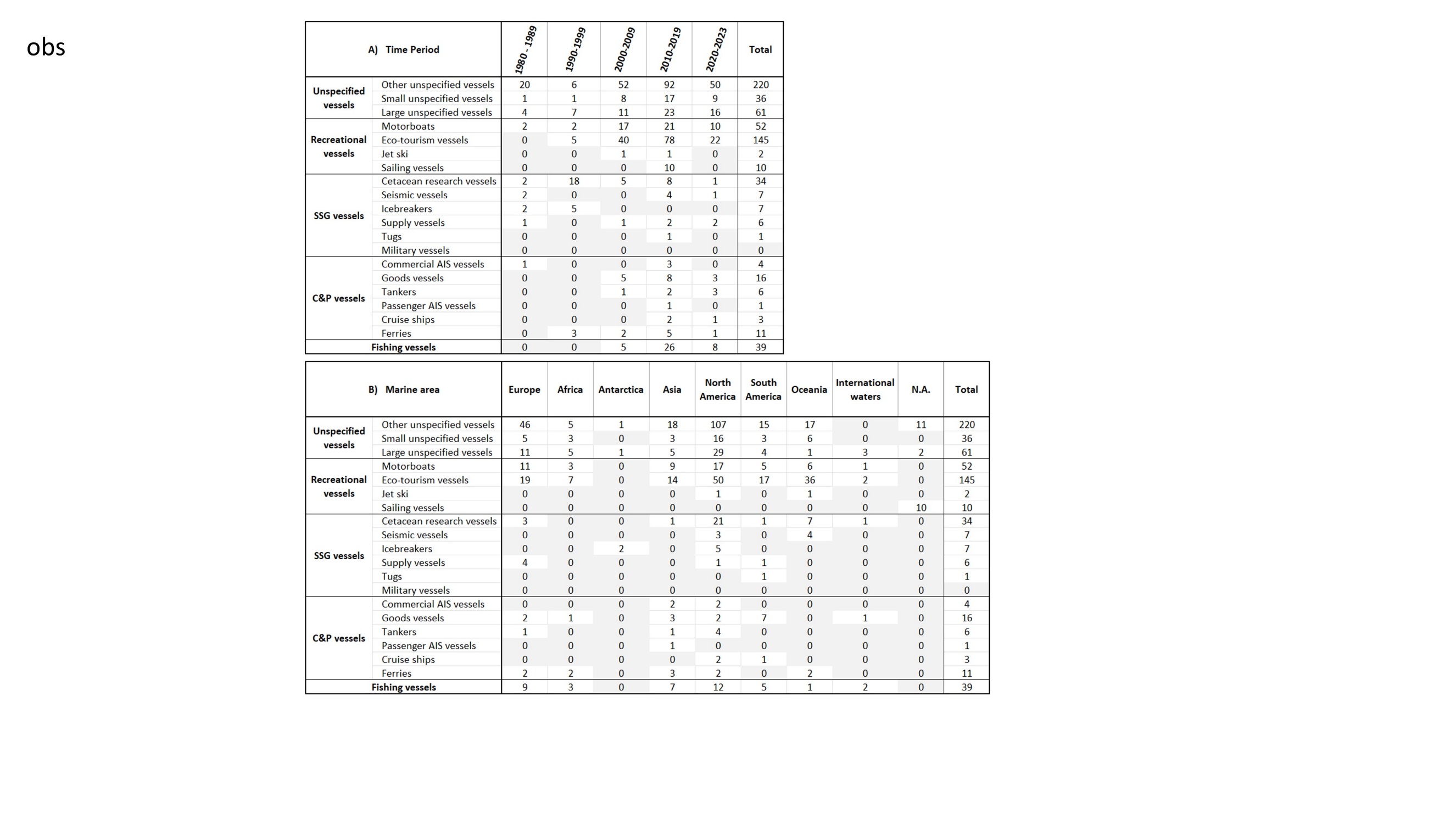


**S6 Table.** Number of empirical records found about different cetacean species for each vessel type. Species with no empirical records are not listed.


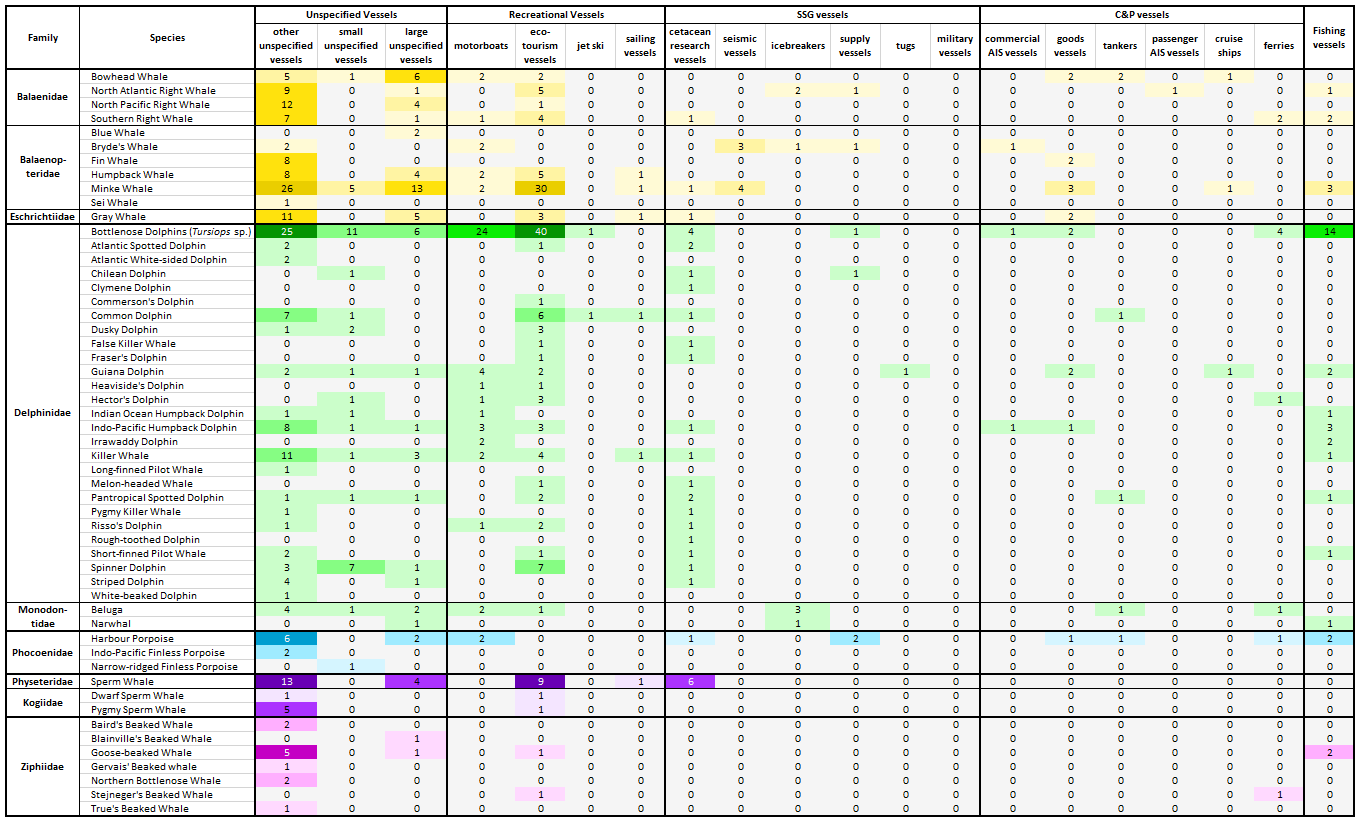


**S7 Table.** Number of predictive records found for each species group for the different time periods (A) and marine areas (B), and number of species studied per year during the different time periods (C).


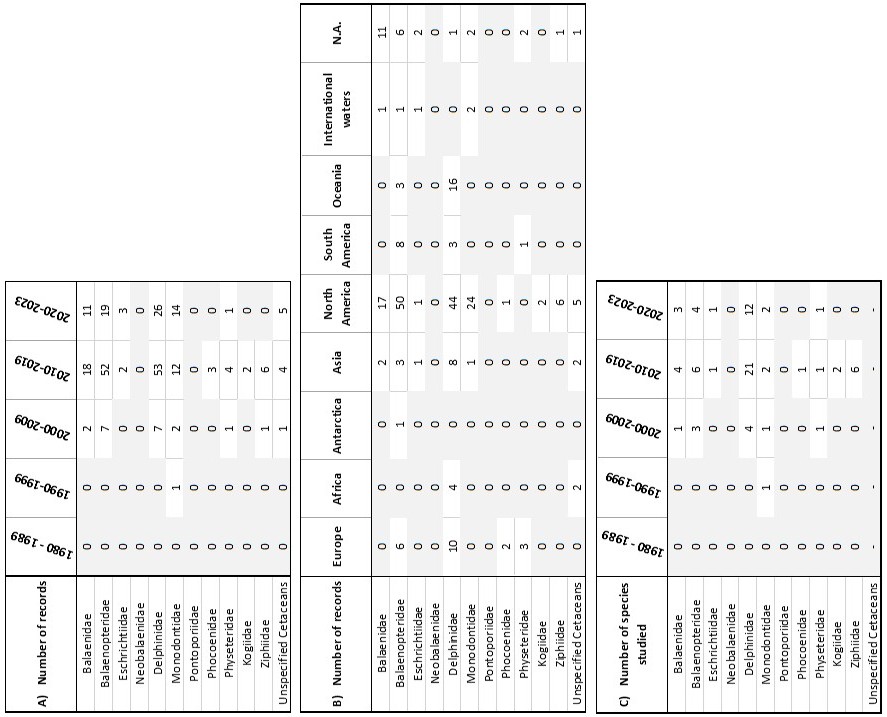


**S8 Table.** Number of predictive records found for each vessel type for the different time periods (A) and marine areas (B).


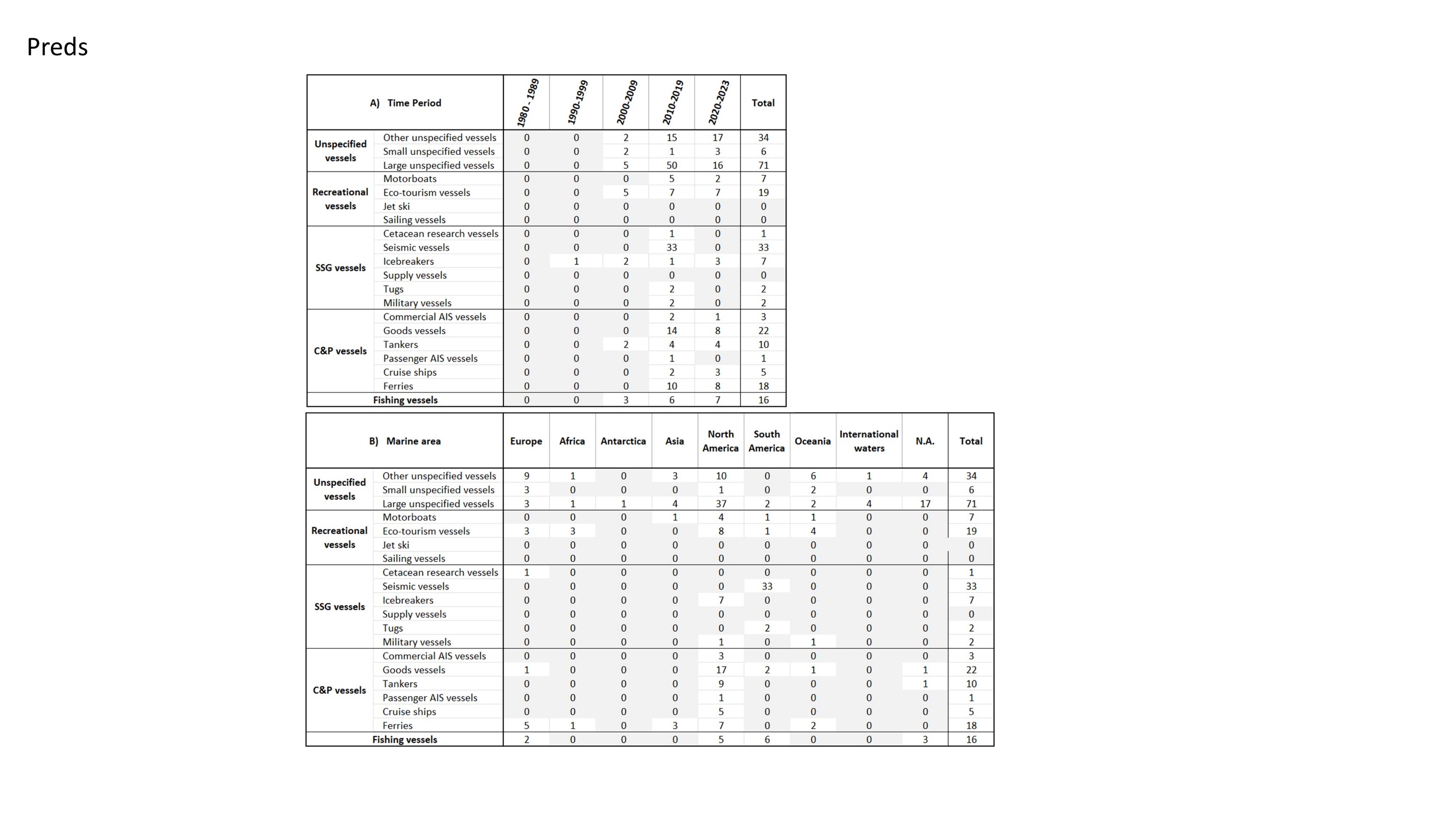


**S9 Table.** Number of predictive records found about different cetacean species for each vessel type. Species with no empirical records are not listed.


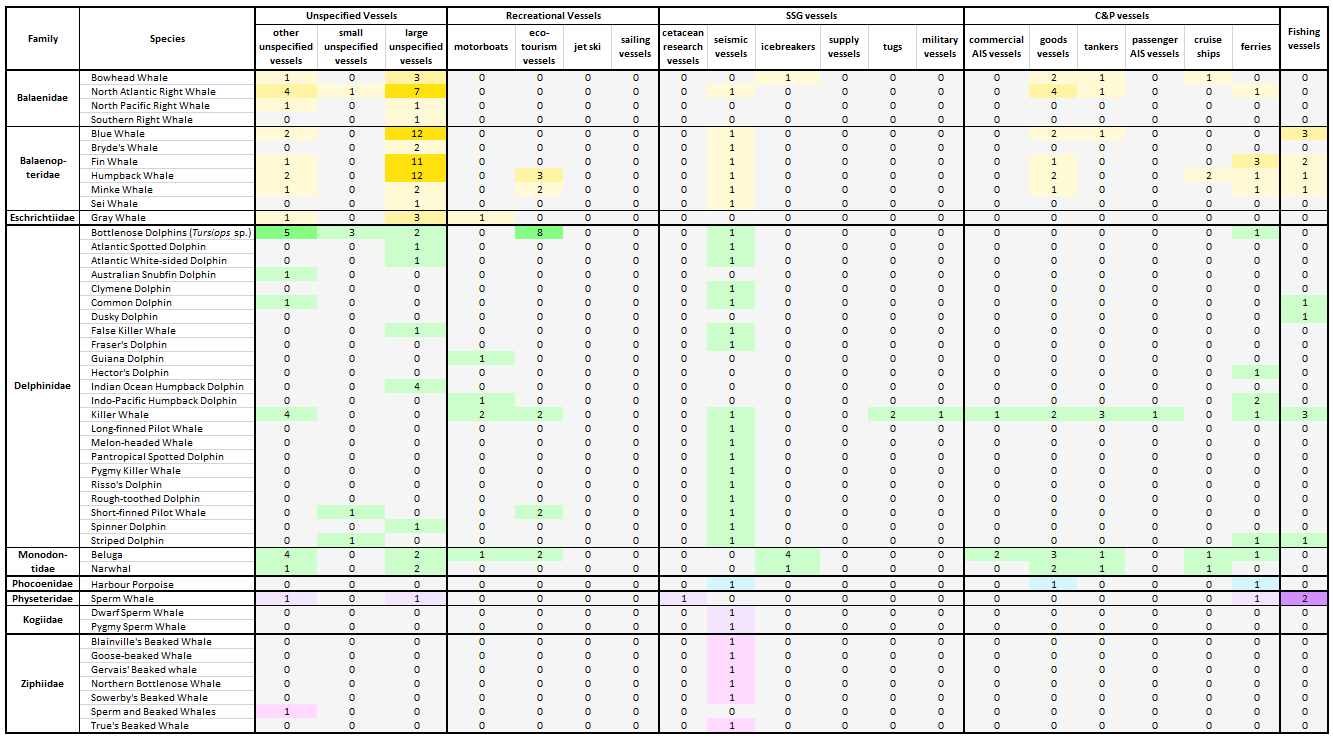


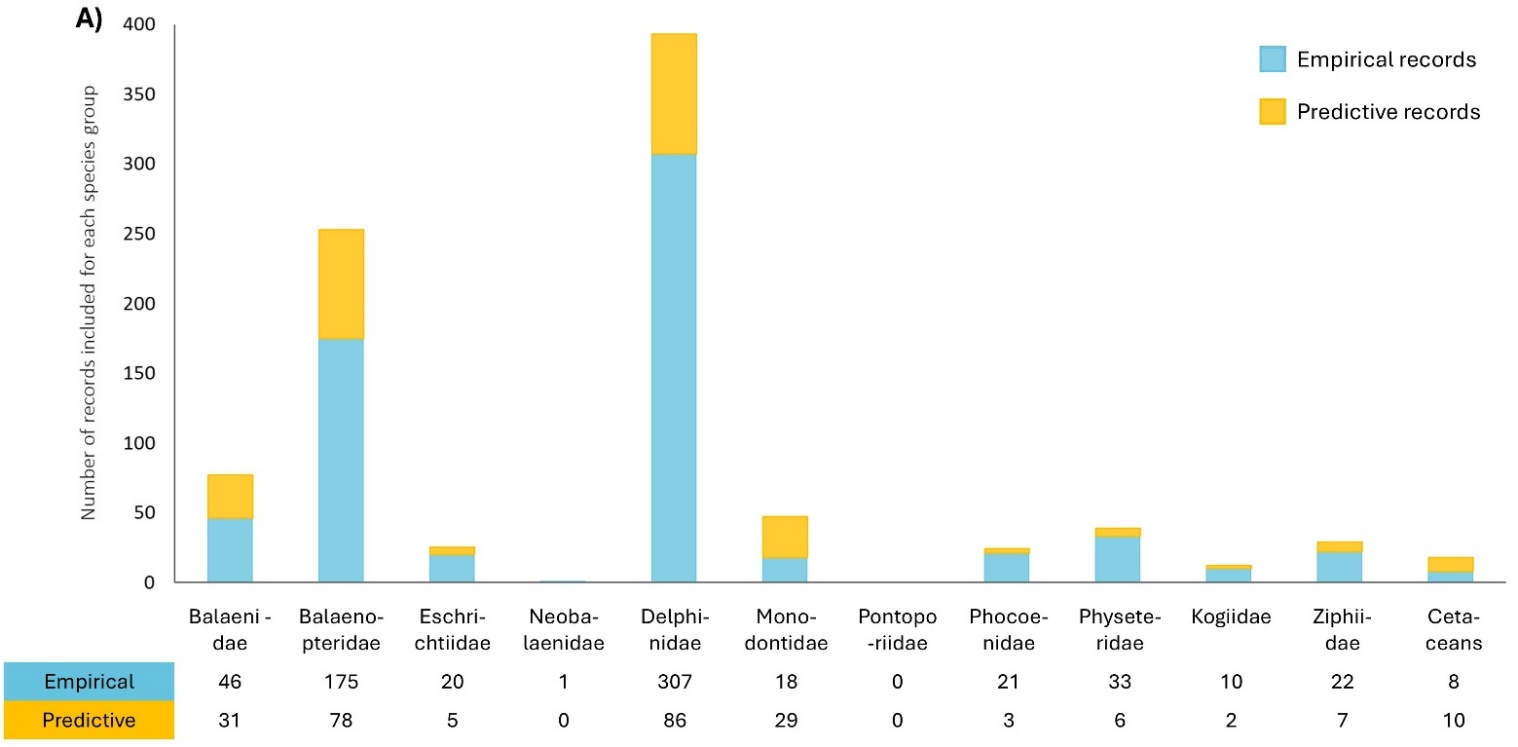

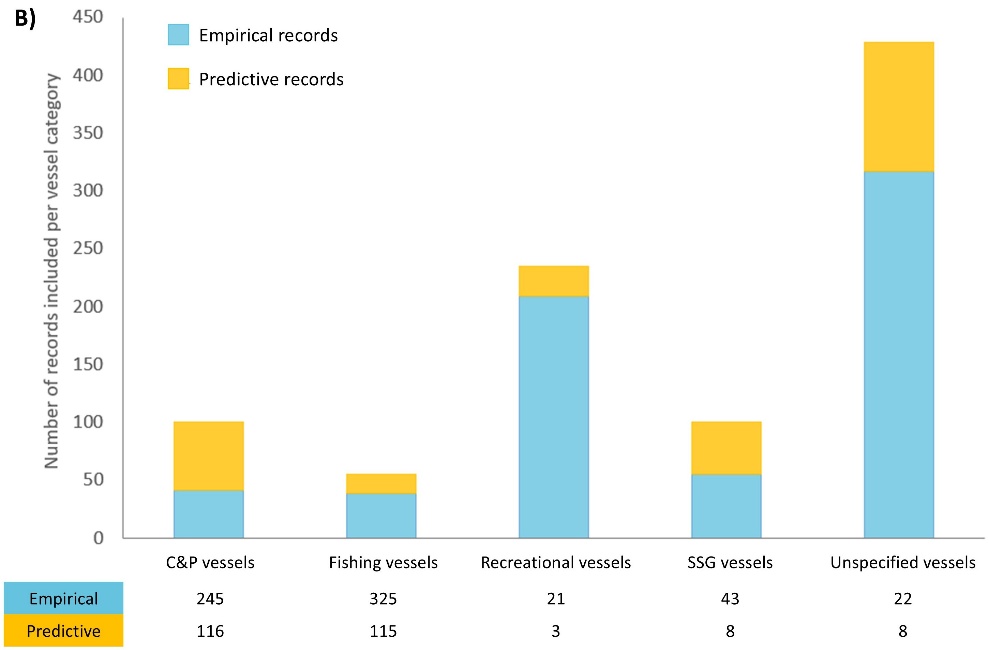


**S1 Fig.** Proportion of empirical (blue) and predictive (yellow) records for each species group (A) and vessel category (B) identified in this systematic map.


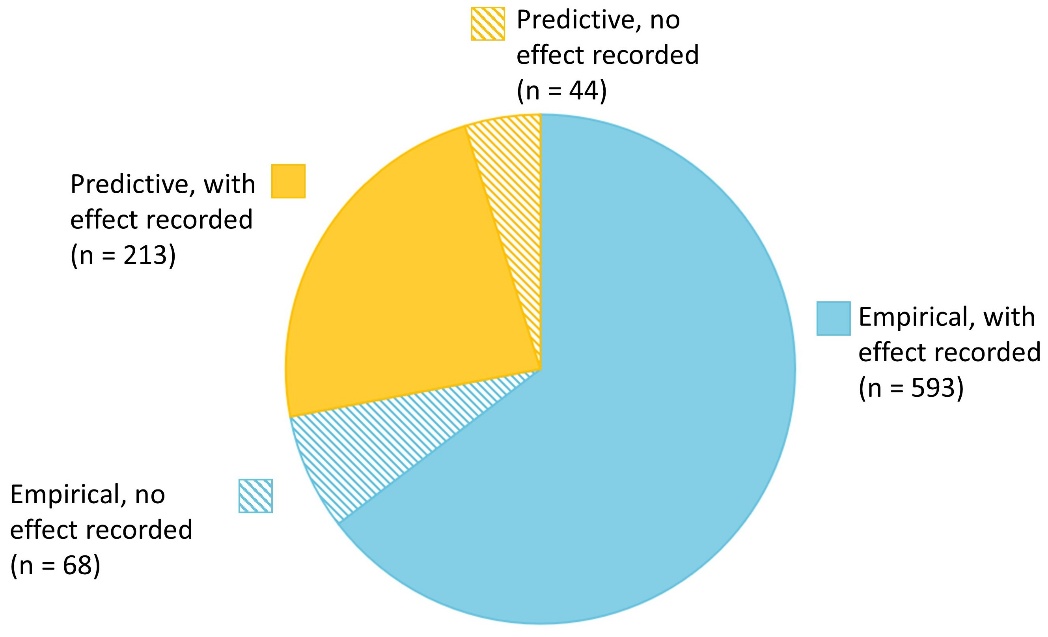


**S2 Fig.** Proportion of empirical (blue) and predictive (yellow) records, and of records that reported a response (effect) from the animal (solid fill) or not (dashed fill).


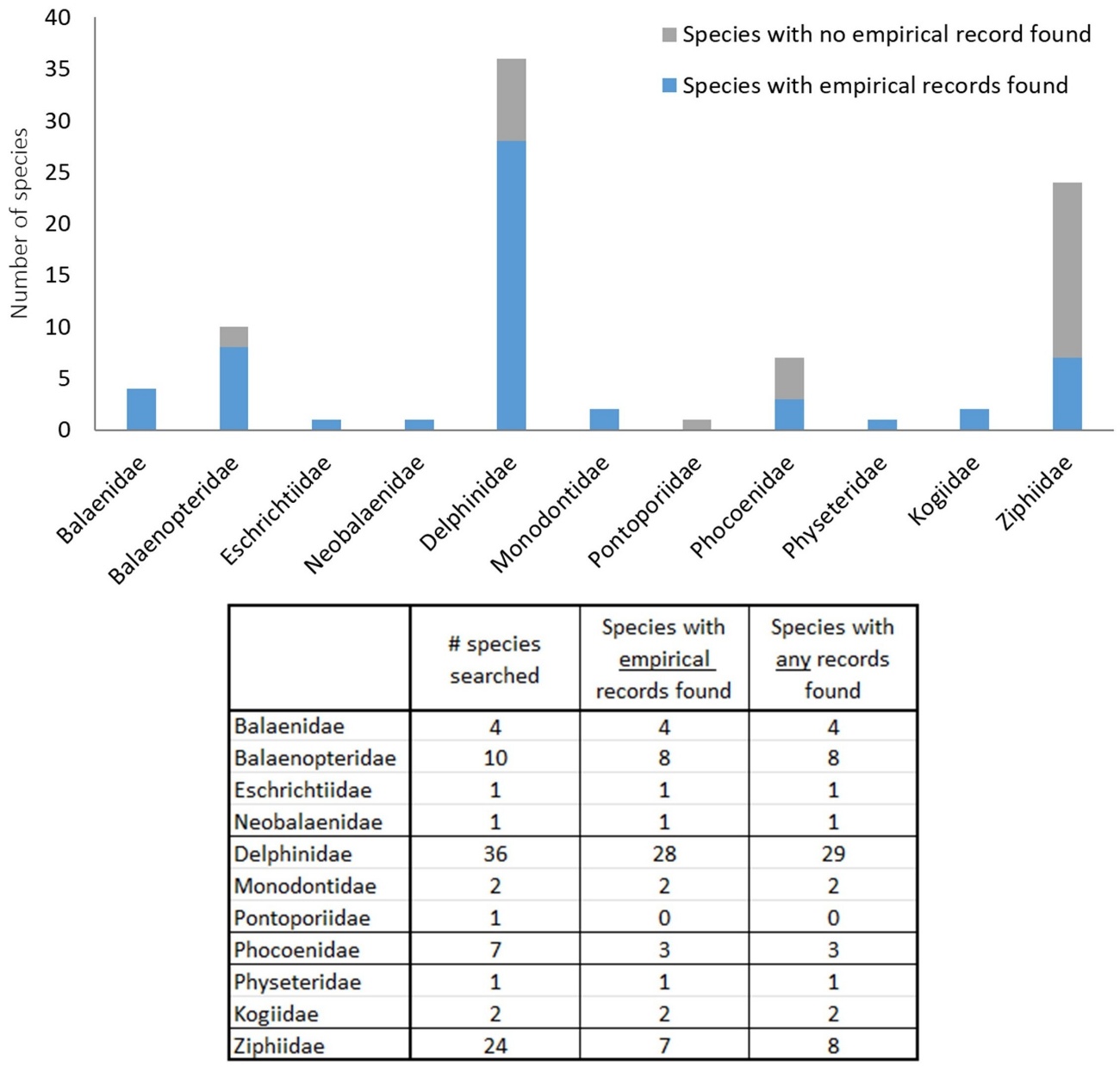


**S3 Fig**. Number of species in each group for which empirical records were present (blue) or absent (dark grey).

**
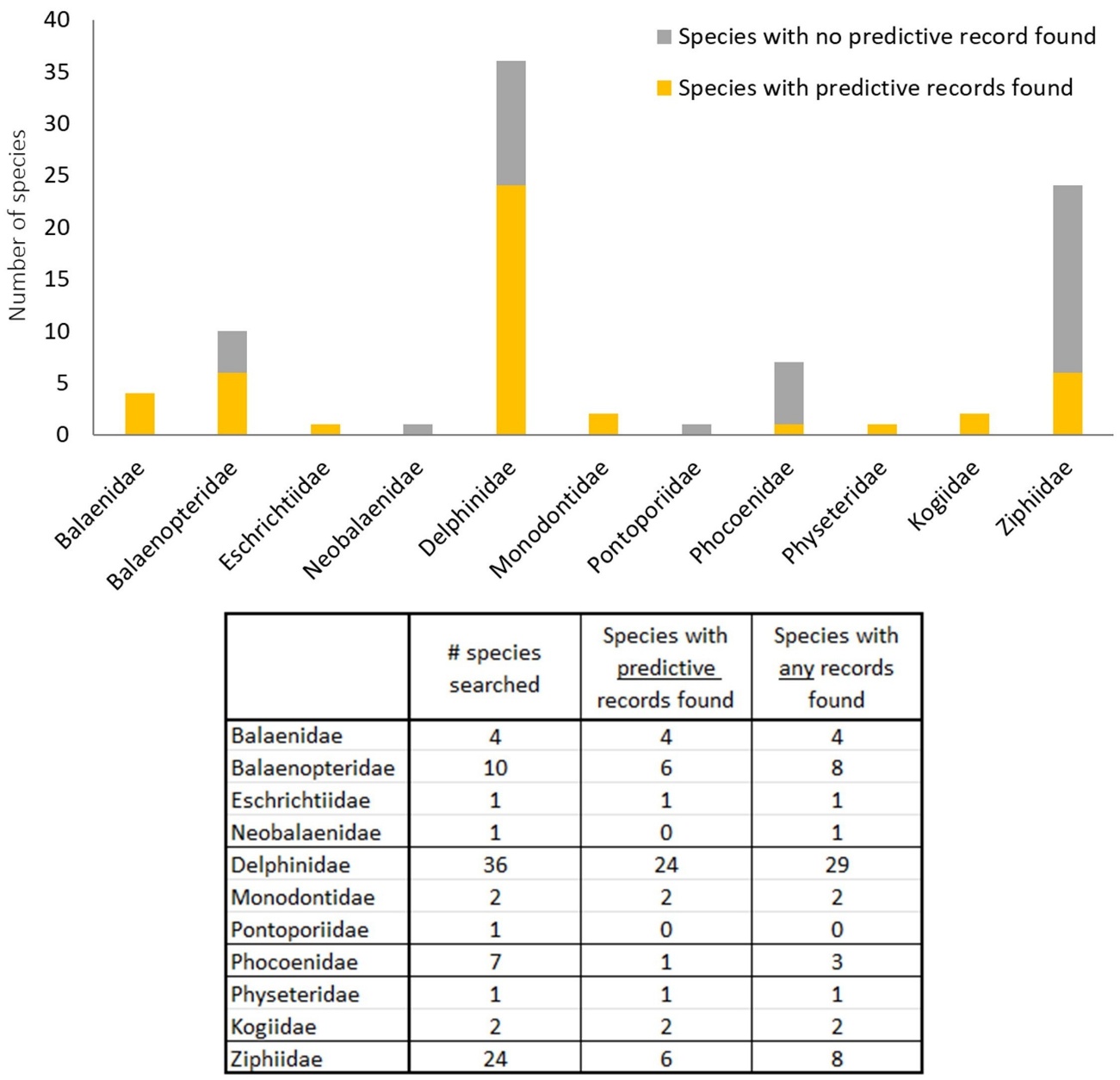
**

**S4 Fig**. Number of species in each group for which predictive records were present (yellow) or absent (light grey).


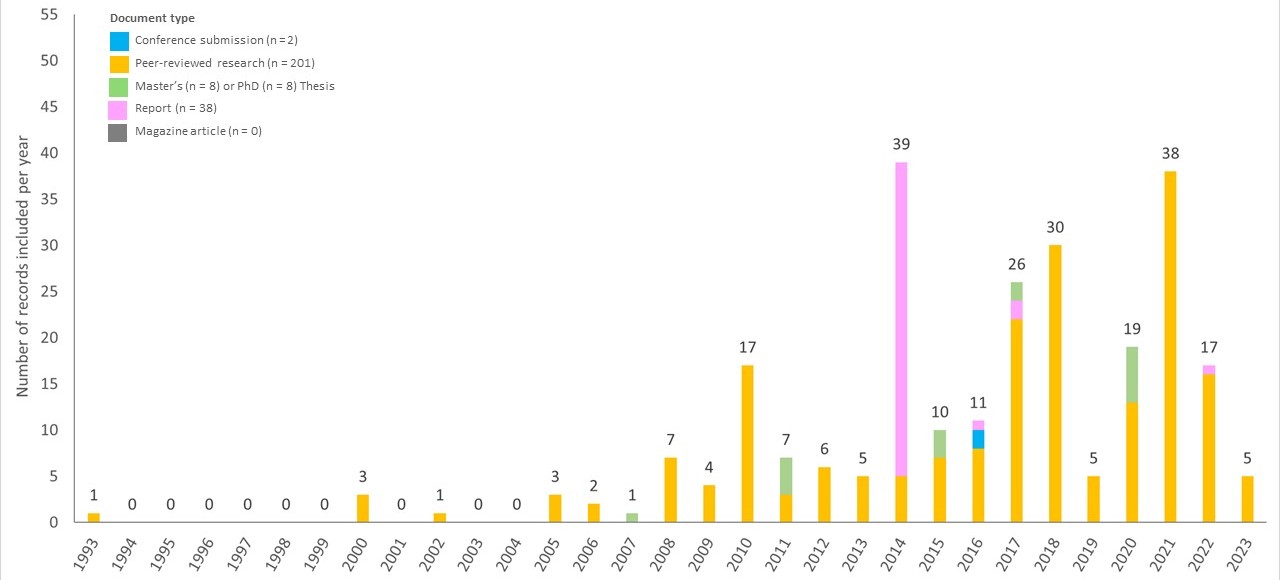


**S5 Fig**. Annual number of predictive records included in this systematic review, with focus on the document type.
