# Supplementary material for: Exploring the evidence of direct threats to cetaceans from maritime vessels: A systematic map
Source: PLoS One. 2026 May 7;21(5):e0348502. doi: 10.1371/journal.pone.0348502 (PMC13152187; doi:10.1371/journal.pone.0348502)
Supplement: Attachment — Submitted filename: Response_to_Reviewers_Cetacean-Vessel_Impacts.docx [file pone.0348502.s004.docx]

27^th^ Feb 2026

**Response to Reviewers RE Manuscript PONE-D-25-59943**

Dear Dr. Rodrigues Paiva, and PLOS ONE,

Thank you for taking the time to consider our manuscript for publication in PLOS ONE. We thank the reviewers for taking the time to provide detailed feedback and considerate comments. We particularly appreciate receiving such constructive suggestions.

We have now addressed all of the reviewers comments and have provided a summary of our responses in the table below, with reviewers comments in **bold** on the left, the authors’ responses in *italics* on the right, followed by any text that has been added (in normal font; with references to the specific lines they can be found at in the updated manuscript). All new additions can also be viewed directly in the updated manuscript (“Revised_Manuscript_Unmarked”). We feel that in addressing the points raised by the reviewers this manuscript has been significantly improved and we would again thank them for their time, effort and careful consideration of this work.

All of the changes we’ve made in response to reviewers comments can be viewed within the ‘Tracked Changes’ document, and a clean document ‘Revised_Manuscript_Unmarked’ is also provided.

Following these changes, we are now resubmitting this updated manuscript for further consideration for publication. We look forward to your response.

Best wishes,

The authorship team

**********************************************************************************

**JOURNAL REQUIREMENTS:**

**3. We note that Figure 2 in your submission contain map images which may be copyrighted. All PLOS content is published under the Creative Commons Attribution License (CC BY 4.0), which means that the manuscript, images, and Supporting Information files will be freely available online, and any third party is permitted to access, download, copy, distribute, and use these materials in any way, even commercially, with proper attribution.**

*Thank you for flagging this omission, the caption for Figure 2 has now been updated to state the source of the layers (all under CC BY 4.0 license).*

**4. Please include a caption for figure 4,5 and 6.**

*This has been addressed and the captions for figures 4 to 9 have also been updated to reflect the new cetacean classification.*

**5. Please include captions for your Supporting Information files at the end of your manuscript, and update any in-text citations to match accordingly. Please see our Supporting Information guidelines for more information:** [**http://journals.plos.org/plosone/s/supporting-information**](http://journals.plos.org/plosone/s/supporting-information)**.**

*We believe this has now been done following these guidelines*

**********************************************************************************

**REVIEWERS’ COMMENTS**

**Reviewer #1:**

| **Reviewer’s Comment** | *Our Response* |
| --- | --- |
| **1) This manuscript addresses an important and timely topic: the impacts of maritime traffic on cetaceans. The authors apply a systematic map methodology to synthesize an impressive body of literature and identify knowledge gaps across species, vessel types, and locations. The topic is highly relevant to marine conservation, maritime spatial planning, and environmental management. The dataset will be a useful resource for researchers and managers. The manuscript is generally well structured, methodologically transparent and clearly written. The methods applied in the review is globally sound and the effort to synthesize predictive tools across studies is appreciated.** | *Thank you for recognising the importance and value of this manuscript and its usefulness for researchers and managers alike.* |
| **2) • in the Introduction with the use of many outdated or inappropriate references, as well as important omissions in the literature,  A major pressure for the marine megafauna is missing here (citation 14): the risk of ship strike. “This growth in maritime activity has also resulted in an increased awareness of the resultant pressures vessels can place on marine environments including various forms of noise, air and water pollution”. Here it is necessary to cite at least one paper per ocean basin: Atlantic, Pacific, Mediterranean, Indian…**  **e.g. 1- for the Caribbean which is a region completely missed in this paper:**  **Bénédicte Madon, Damien Le Guyader, Jean-Luc Jung, Benjamin De Montgolfier, Pascal Jean Lopez, Eric Foulquier, Laurent Bouveret, Iwan Le Berre, Pairing AIS data and underwater topography to assess maritime traffic pressures on cetaceans: Case study in the Guadeloupean waters of the Agoa sanctuary, Marine Policy, Volume 143, 2022, doi.org/10.1016/j.marpol.2022.105160.**  **2- for the Med: Maxime Sèbe, Léa David, Frank Dhermain, Sophie Gourguet, Bénédicte Madon, Denis Ody, Simone Panigada, Hélène Peltier, Linwood Pendleton, Estimating the impact of ship strikes on the Mediterranean fin whale subpopulation, Ocean & Coastal Management, Volume 237, doi.org/10.1016/j.ocecoaman.2023.106485.**  **A citation to avoid citing only papers in the Pacific (with citations 21-22 and/or with the new group in place of citation 14):**  **Fumagalli, M., Cesario, A., Costa, M. (2019). Where Dolphins Sleep: Resting Areas in the Red Sea. In: Rasul, N., Stewart, I. (eds) Oceanographic and Biological Aspects of the Red Sea. Springer Oceanography. Springer, Cham. https://doi.org/10.1007/978-3-319-99417-8_17**  **In the group 58-61: this reference should be added: Schaffar A, Madon B, Garrigue C, Constantine R (2013) Behavioural effects of whale-watching activities on an Endangered population of humpback whales wintering in New Caledonia. Endang Species Res 19:245-254 https://doi.org/10.3354/esr00466**  **For “Recent advances in technologies such as passive 121 acoustic monitoring (PAM) and remote sensing (“Automatic Identification Systems (AIS) data) have advanced our understanding and ability to evaluate and in some cases mitigate the threats posed by vessels” previous proposed citation should be used as “e.g.”: Bénédicte Madon, Damien Le Guyader, Jean-Luc Jung, Benjamin De Montgolfier, Pascal Jean Lopez, Eric Foulquier, Laurent Bouveret, Iwan Le Berre, Pairing AIS data and underwater topography to assess maritime traffic pressures on cetaceans: Case study in the Guadeloupean waters of the Agoa sanctuary, Marine Policy, Volume 143, 2022, doi.org/10.1016/j.marpol.2022.105160.** | *Thank you for pointing out these issues and for providing examples of other references we could use. Citations have now been updated throughout the whole manuscript, making sure to include several more relevant and up-to-date publications and to expand the variety of locations they refer to, where possible.* |
| **3) The introduction could better articulate why a systematic map (rather than a systematic review or meta-analysis) is the appropriate tool: what specific decisions or management needs does a map support?** | *In order to highlight why a systematic map was chosen rather than alternative approaches, we have added the following text in blue (see lines* ***142-146****):*  Previous reviews have focussed on describing the effects of specific type of impact [144] or quantifying evidence available for a particular species or group of recipient species [12], vessel type or activity [12,32], or defined geographic areas [145]. Few, however, have taken a holistic approach, assessing global literature on marine vessel impacts across all vessel types and cetacean species. This study aims to, for the first time, synthesise the currently available evidence for all marine cetaceans, all vessel types and all geographies, allowing for a fuller understanding of this issue. Given the large and highly varied body of literature on this topic, a systematic map was used, over a more extensive literature review, in order to better highlight knowledge gaps and quantify the spatial distribution of the evidence. |
| **4) I do not agree with this: “While some impacts (like collisions between cetaceans and vessels) are limited to the exact location of the vessels [45],”, the wording as is now, confuses between where the event happens vs where the ecological impact manifests, please rewrite to clarify.** | *Thank you for pointing this out. We have now re-worded the sentence to hopefully improve its clarity (see line* ***82-89****):*  While direct interaction between cetaceans and vessels may be localized, the wider ecological impacts are often experienced and measured elsewhere. For example, although vessel strikes occur at the specific location of the vessel [71], demographic changes, declines in population and ecosystem consequences are manifested and detected far from the site of collision [72–75]. And other vessel-related threats (like underwater noise generated by vessels) have a much broader spatial footprint, with the potential to affect cetaceans over greater distances, with the magnitude of responses depending on species sensitivity, habitat characteristics, and source levels [76–78]. For example, humpback whales reduced the number of vocalizations when vessel traffic passed within 1.2km away from the animal [79], Blainville’s beaked whales (Mesoplodont densirostris) altered their feeding behaviour in response to vessel noise up to distances of 5km [35], and harbour porpoises (Phocoena phocoena) showed avoidance responses up to 4km away from construction-related vessels [80]. |
| **5) • In the Results, several areas require clarification, correction with taxonomic inconsistencies and errors in figures**  **Latin names of species are missing. Please do not cite only generic names but use Latin ones also.**  **Fig.5 to 8: The classification in the figure lacks consistency, as it juxtaposes taxonomic ranks from different hierarchical levels—such as the suborder Mysticeti, the superfamily Physeteroidea, and families like Phocoenidae and Ziphiidae—alongside the informal grouping 'Delphinidan'. This undermines taxonomic coherence and comparability.** | *To address this comment, species are now grouped by family and latin names are added in the first instance where a species is referred to.* |
| **6) Fig.9 is wrong. Where are vessel types?** | *Thank you for highlighting this error in the y-axis, it has now been fixed.* |
| **7) S5 Fig: Annual number of predictive records included in this systematic review, with focus on the document type. Without years 1981-1992, should be redone removing years 1981 to 1992.** | *This has now been updated.* |
| **8) Figures could be more interpretive—e.g., maps showing density of studies relative to global shipping intensity.** | *Agreed, being able to compare study density to global vessel traffic intensity would be incredibly useful. Within the paper, the location of the study has been recorded as the centre of the area, rather than representing its full extent, because many documents didn’t provide clear maps or coordinates, or the exact location was unclear (for example, in the case of stranding reports). We therefore feel that using this data would be inappropriate to compare study density and vessel traffic intensity, and may lead to misleading outputs. In addition, accurate maps on vessel traffic may not be available at a global scale for all vessel types, especially the typically non-AIS “recreational” categories used in this review. However, we acknowledge this caveat to interpretation and use of the data, and have added additional text (see comment 13).* |
| **9) In the results section, the authors do not explore publication bias, research capacity disparities or the influence of regulatory frameworks.** | *We appreciate the reviewer highlighting these gaps, and the following text (highlighted in blue) has been added to the Discussion (see line* ***469-492****):*  The results can therefore help shed light on possible geographical disparities in the research effort on vessel impacts, with most of the evidence being gathered in North American or European waters and far fewer records documenting impacts in international waters or other marine areas. This could be partially explained by the fact that this study reviewed only documents written in English: certain document types (like technical reports, thesis or grey literature) will likely be published in the country’s main language and not always translated into English, which may have resulted in records from non-English speaking areas being under-represented in the results. Other factors that may have played a role in the geographical differences in the evidence base include the dominance of the Western scientific paradigm in research, funding availability, policy and conservation obligations, as well as challenges associated with surveying expansive or remote areas [185]. The observed variability in research outputs could also reflect a global disparity in monitoring capacity [182]. Countries with greater access to information gathering technologies (e.g. dense/accessible AIS coverage, established Passive Acoustic Monitoring (PAM) networks, repositories of satellite imagery or the capacity to task satellite time, and long-term, funded, research programmes) are better equipped to generate empirical evidence, whereas regions with limited resources or a lack of established research infrastructure may struggle to implement comprehensive monitoring programs, leading to significant knowledge gaps concerning local cetacean populations and the potential impacts of maritime vessels [183-185]. Areas with greater evidence (e.g., North American/European waters) are also where management tools, such as those implemented by the International Maritime Organization (IMO), including traffic separation schemes and areas to be avoided, or by local port and harbour authorities, such as seasonal or dynamic vessel speed regulations, and emissions/noise controls, are most frequently trialled or implemented [186-188]. In addition, the presence of regulatory frameworks in high‑income countries (such as EU Habitats and Birds Directives, the Natura 2000 network or the U.S. Marine Mammal Protection Act) have directly stimulated investment in cetacean monitoring and research [189-192], whereas in regions of the global South, weaker implementation capacity and competing development priorities have limited sustained cetacean research despite legal protections [193]. All these differences could create a feedback loop where management policies and regulatory frameworks reflect monitoring capacity rather than true need based on impact risk, underscoring the importance of international cooperation for targeted capacity building and technology transfer [194-196]. Lastly, there may be other safety obstacles (such as piracy) that could hinder research and possibly affect the number of other maritime vessels on the water, especially in the global South [197-198]. |
| **10) The distinction between empirical and predictive studies is useful but the manuscript does not analyze how these two evidence types complement or contradict each other.** | *The comparison was not within the scope of the paper, however we fully agree that such comparison would be interesting to explore further, and have added it as a recommendation for future studies (text included as part of the response to comment number 20; see lines* ***746-750****). The choice to present only empirical studies was made to showcase how much research is actually available from hard evidence and to avoid giving a possibly inflated sense of knowledge by including modelling studies, which were therefore only presented in the Supplementary Materials.* |
| **11) The use of record counts may disproportionately reflect heavily studied systems or species. In particular, a single long-term study contributing multiple records could inflate the perceived strength of the evidence. This issue is not explicitly or adequately addressed.** | *Great point. We have now addressed this further in the “Cetacean Species” section of the Discussion (see line* ***527-551****):*  Lastly, it is important to acknowledge that, in using record counts as a measure of research effort, the values presented could be influenced by long-term studies or establishment of research programmes which can result in multiple publications/records being generated. While the size of the marine regions used to collate records in this systematic map does not allow us to evaluate the extent of fine-scale spatial patterns in research efforts, some of the geographical differences that were, identified for individual species may be, at least in part, also related to the presence of such long-term studies or established research programmes. For example, nearly half (15/32) of the European studies on Atlantic bottlenose dolphins (Tursiops truncatus) were documented within UK waters. Similarly, virtually all of the identified records for killer whales (22/24) were collected in North American waters, there were no records available for Antarctic, African, European and Asian waters, despite this species having a global range. Of these North American records, majority focus on Southern Resident killer whales (e.g. [215-218]), while less research has focused on other populations, such as Northern Resident or transient killer whales (e.g. [219-220]). This suggests that research effort may not be evenly distributed between populations, not just species, as highlighted by another study that considered evidence of vessel impacts for specific species subpopulations in the Arctic [71]. Because cetacean populations differ in distribution, habitat use, demography, and baseline status, the same vessel pressure may present very different risk(s) across sub‑populations [221,222]. Spatially averaged or species‑wide assessments ignore local hotspots of high overlap, place‑specific sensitivities (e.g. small, closed, or breeding populations), demographic differences in response, and variation in traffic type and seasonality [222-227]. As such, these findings suggest that just because there appears to be a relatively high volume of available evidence for a species, this shouldn’t be misinterpreted, or preclude from further efforts to investigate possible variability in exposure and responses at the population or sub-population level. |
| **12) • In the Discussion where strengthening, or deeper critical interpretation are needed. Some methodological choices need justification and the discussion could more explicitly connect findings to management implications, biases in the literature, and future research priorities.**  **All impacts are treated equally (e.g. brief behavioral response vs lethal strike). This is acceptable for a map but care should be given to avoid misinterpretation of record frequency as impact severity.** | *Agreed. To emphasise and explicitly acknowledge this point we’ve added the text highlighted in blue to the discussion (see line* ***660-663****):*  Lastly, it is important to note that the type of impacts considered in relation to each vessel type requires further scrutiny. Research effort might not be equally distributed across impacts, and indeed we noted that, for example, effects of behavioural disturbance from recreational motorboats appear to be more studied [253–257] than those of noise [145,258]. Importantly, this systematic map does not analyse or consider different types of impacts separately or based on their severity, and as such, record frequency cannot, and should not be interpreted as a proxy for the scale or severity of the threat that a vessel type might pose to a particular species. |
| **13) There is no discussion on how spatial assignment may influence results: using the “centre of the study area” may distort representation for large study regions (e.g., migratory corridors, ocean basins). The authors should discuss potential spatial bias introduced by this simplification.** | *We appreciate the reviewer highlighting this point, in fact we took the decision to treat the data in this matter to avoid misrepresenting the spatial accuracy of the data and we did not explain this sufficiently. To address this we have added the following (highlighted in blue) to the Discussion section (see line* ***452-453*** *and* ***493-498****)*  Spatial trends are more difficult to infer. Larger regions may hold a higher number of records partly because of their size, and not necessarily only because of differences in research effort. Similarly, vessels may be unevenly distributed between marine regions, but accurate estimates on the number and types of vessels operating in each area are lacking, as the current reporting system appears to focus more on the country of registration rather than place of operation [2]. However, information on cetacean presence is more readily available and numbers are indeed comparable between the marine regions as defined for this systematic map (45-54 species, [180]). The results can therefore help shed light on possible geographical disparities in the research effort on vessel impacts, with most of the evidence being gathered in North American or European waters and far fewer records documenting impacts in international waters or other marine areas.  It’s finally important to note that in this systematic map a “central location” was chosen to indicate where the research has been conducted, as often the information on the full extent of the study area was missing or unclear, and it could vary the different types of impacts being studied. While this standardised approach allowed to place a study within the respective marine region, it does not allow to discern finer scale patterns within these regions or where the exact study locations are in relation to high vessel traffic areas. |
| **14) The discussion needs to be expanded and strenghtened. As such, it does not sufficiently connect findings to:**  **• management needs (e.g., IMO guidelines, MPAs, vessel speed regulations),** | *Thank you for suggesting these additional discussion points, the authors agree and feel that by adding and highlighting these connections in the discussion that these strengthen the potential impact of this study's findings. Please find below the specific text (highlighted in blue) exploring these points that have been added to the discussion (see lines* ***556-559****):*  This map has shown that whilst interaction with commercial cargo ships and passenger vessels is recognised as a potential threat related to cetacean conservation [134,228], there is less empirical evidence than expected documenting their impacts [71], with only 41 out of 100 records providing empirical evidence. This gap is especially critical from a management perspective, as these are the target vessel categories of many regulatory frameworks, from the international IMO guidelines to national regulations, such as traffic separation schemes, speed-reduction measures and routeing changes [32,229]. |
| **15) • technological advances (AIS, PAM, satellite tracking),** | *The following text (highlighted in blue) was added to the Discussion to explore this topic (see lines* ***635-655****):*  Despite the challenges in identifying and categorising vessel types involved in some records, this review can highlight specific knowledge patterns. In contrast to the lack of empirical evidence on fishing, sailing and commercial cargo and passenger vessels, there are records to suggest the presence of potential impacts associated with cetacean research activity itself, though effort appears to have focused mostly of dolphin species (Delphinidae and Monodontidae). While the recent years have seen a rapid growth in the use of remote sensing and autonomous technologies to quantify and mitigate vessel impacts on cetaceans [242–244], as long as boat-based research will be conducted it would still be valuable to assess the potential impact of the research platform itself, especially on those species groups for which evidence is currently lacking. It is also true that unmanned aerial vehicles (UAVs), autonomous surface and underwater vehicles, and satellite remote sensing are increasingly being combined with PAM and biologging to map cetacean distribution, behaviour, and exposure to shipping noise and collision risk, with higher resolution and at lower cost than traditional ship‑based surveys, thereby providing an emerging technological toolbox for evidence‑based vessel‑impact assessment and mitigation [245-247]. For example, passive acoustic monitoring (PAM) has moved beyond traditional moored recorders to include cabled observatories, seafloor telecommunication cables, and mobile platforms (such as underwater gliders, wave gliders, and other unmanned surface vehicles), greatly expanding spatial and temporal coverage of ship noise and whale occurrence [247, 248]. These mobile PAM systems are also now being utilised in dynamic management schemes, where near‑real‑time detections of endangered North Atlantic right whales from gliders trigger mandatory vessel slow‑downs and other risk‑reduction measures over large regions and across seasons [249-250]. In parallel, satellite‑linked telemetry of whales is increasingly combined with terrestrial and satellite AIS data on vessel movements to quantify spatial overlap, identify critical habitats and migratory corridors that intersect high‑traffic routes, and evaluate ship‑strike risk at regional and basin scales [244,251,252]. |
| **16) • global heterogeneity in monitoring capacity.** | *The text exploring this point has been added to the discussion (see lines* ***469-492****) and has been incorporated as part of the response to comment number 9.* |
| **17) In its current version, the discussion overlooks major opportunities to strengthen the relevance and impact of this work, particularly by failing to articulate implications for methodological standardization.**  **The authors note that vessel heterogeneity is poorly captured in the literature but do not propose concrete solutions (e.g., standardized reporting frameworks, integration with AIS metadata).** | *Please find below the specific text exploring this point that have been added to the discussion (see lines* ***715-733****):*  It is however important for this new research to follow more standardised protocols. The heterogeneity in methodologies identified across studies highlights the need for greater standardisation in data collection, reporting and vessel classification. While for some stressors (e.g. noise) vessel types have been well characterised and responses to different vessel types are well studied [283], such level of detail may not be available for other stressors. Only recently, risk of lethal collision with large whales has been described for vessels of different sizes, while previously it was solely based on vessel speed [238]. And for other impacts, like behavioural disturbance due to vessel presence, variation in responses to different vessel types have not been analysed yet and the vessel categorization systems used for noise (by type and operational characteristic) and collision (by size) may not be suitable to assess this impact as a variable relating to activity might need to be incorporated to account for those vessels that are more likely to encounter cetaceans due to their operations (e.g. whale-watching and fishing vessels) or those that have the ability to actively pursue the animals (e.g. whale-watching or recreational boats, as opposed to commercial vessels with stricter routes and schedules). Despite these challenges, developing shared protocols for describing vessel characteristics and defining vessel categories according to size and activity, and developing consistent monitoring approaches would enhance comparability across studies and regions, improve the transferability of results to management contexts, and facilitate the integration of evidence into policy and regulatory frameworks*.* |
| **18) The manuscript acknowledges that more than 40% of records could not be assigned a vessel type. For unspecified vessels, “expert judgement” was used to infer vessel size/type. This introduces non-trivial classification bias, particularly given the high proportion of records lacking vessel-type attribution and and the central role of vessel type in the presented results. This is a major limitation and the authors do not sufficiently interrogate why this occurred: was it due to poor reporting in primary studies or limitations in the classification scheme? Moreover, the use of expert judgement to infer vessel type when not explicitly stated introduces subjectivity: How was consistency ensured? Was a second reviewer consulted for ambiguous cases?** | *Thank you for raising this and we agree and are able to provide further transparency relating to this process and the steps taken to minimise classification bias as much as possible. Further details on the possible motivations for the high number of unspecified vessels is now provided in the Discussion (see lines* ***605-623****):*  One of the notable challenges with this review was classifying the records into different vessel types, as very often (in almost 40% of cases) studies did not include specific details on the vessels included in the research. In many studies authors often referred to the stressor as simply “vessels” and no further information on vessel characteristics could be inferred from the text. This was most common for those studies considering the effects of underwater noise, when the source vessel was not specifically identified or cumulative noise exposure was being evaluated. Similarly, reports of stranding events or injuries on the animals were rarely able to explicitly state the size and type of vessel involved, because it was often unknown. There were cases, however, where the terminology used or the location of the study area allowed us to make informed decisions about the size of these “unspecified vessels”. For example, documents referring to “commercial traffic” or “shipping traffic”, or those evaluating noise inside shipping lanes were categorised as “likely-large unspecified vessels”, as they would likely target large cargo or passenger vessels but didn’t explicitly state so. Similarly, studies conducted in coastal waters (3-5 nautical miles from the coast) and away from main ports were assigned to the “likely-small unspecified vessels” category, as they probably referred to smaller recreational boats. While these decisions were discussed between the authors in an effort to ensure consistency, we acknowledge that the use of expert judgement introduces a level of subjectivity in the classification and that many of these studies including “unspecified vessels” could still refer to vessels of various sizes and types. |
| **19) While mentioned, cumulative impacts (e.g., traffic density + noises) are not fully explored.** | *Thank you for the suggestion. The following text has been added to the Discussion (see lines* ***664-681****):*  Lastly, it is important to note that the type of impacts considered in relation to each vessel type requires further scrutiny. Research effort might not be equally distributed across impacts, and indeed we noted that, for example, effects of behavioural disturbance from recreational motorboats appears to be more studied [253–257] than those of noise [145,258]. Importantly, this systematic map does not analyse or consider different types of impacts separately or based on their severity, and as such, record frequency cannot, and should not be interpreted as a proxy for the scale or severity of the threat that a vessel type might pose to a particular species. Beyond individual impacts there is a growing body of evidence pointing to the necessity of considering the cumulative impacts that vessels may have on cetaceans, when investigating the conservation concerns that these stressors may have [259–262]. Approaches that attempt to take a more holistic view of impacts, such as Cumulative Impact Assessments (CIAs) and Ecosystem-Based Models (EBMs), can provide a more inclusive framework for trying to evaluate the combined effects of diverse maritime activities on marine mammal populations [263,264]. However, attempting to evaluate multiple stressors and their interactions over space and time to provide a more realistic understanding of anthropogenic pressures is something that is widely pointed to as being one of the biggest challenges when managing marine systems [265-266]. This comprehensive perspective is essential for informing policy development and it underscores the urgent need for standardized methodologies in impact assessment and monitoring [261,267]. Indeed, few of the documents identified from this review attempted to consider multiple vessel types (e.g. [51,257,268,269]) and none explicitly assessed the cumulative impacts from all those vessels. Similarly, studies that do attempt to evaluate cumulative exposure tend to focus on a single type of stressor (e.g. noise) and often examine different sources in addition to vessels, rather than multiple vessel types [270-272], while impacts from different stressors are understudied and empirical evidence is especially lacking [273]*.* |
| **20) Finally, the manuscript could better articulate how this systematic map can guide:**  **• future systematic reviews,** | *Thank you for the suggestions. We have now added text to explain this further (see lines* ***734-761****):*  In addition to guiding new empirical research, this systematic map can also guide future systematic evidence analysis. Firstly, future synthesis should account for different responses from the animals, with clear distinctions between behavioural, social or physiological responses, vocalization changes, and lethal or sublethal injuries, as well as short- or long-term consequences. Secondly, future reviews should explore seasonal patterns in the available evidence and the possible lack of data during months where survey conditions are commonly unfavourable (like boreal winter, austral summer, monsoon seasons). High-resolution spatial patterns should also be investigated to assess possible biases at a local scale and between inshore and offshore waters, which necessitates recording the full extent of the study area rather than a central location. Thirdly, with the emergence of new technologies, future synthesis could include information of the data sources used for both cetaceans (e.g. land- or ship-based studies, visual or acoustic, eDNA) and vessels (e.g. land- or boat-based surveys, AIS data), with particular interest on temporal and spatial patterns. It will also be important to compare distribution of empirical and predictive evidence, as that is likely to differ between vessel types, species (or species groups) and response types. For example, research using AIS data might be more predictive in nature, and studies on elusive species might rely more strongly on modelling due to the lack of observational records. At a smaller scale, future systematic literature reviews should focus on assessing potential knowledge gaps at the population and sub-population level for those apparently better-studied species, like humpback whales, bottlenose dolphins, killer whales and sperm whales. For this to happen, a higher spatial resolution than the one used in the current study is needed. In particular, extracting information of the size on the study area (rather than the centre location) would allow to better evaluate overlap with both high vessel traffic zones and protected areas, to assess whether cetaceans are being studied and protected where they are actually more vulnerable. In addition to systematic literature reviews, future studies focusing on data-deficient species or vessel types should also attempt to collate information for any types of documentation and media (including generally disregarded sources like blog posts or videos) as well as conducting interviews with marine users, like has been done to document entanglement and bycatch for example [284–288]. |
| **21) • targeted field studies,** | *To address this comment, the following text has been added to the Discussion (see lines* ***693-714****):*  Recognizing the differences in species-specific responses and vessel characteristics is critical for tailoring conservation policies and monitoring strategies effectively. Different cetacean species exhibit varied sensitivities to vessel disturbances [256,276,277], and vessel types differ substantially in their size, speed, noise frequency, and operational patterns, which may result in different impacts [278,279]. This heterogeneity necessitates conservation approaches that are nuanced and adaptive, ensuring that mitigation measures are both species-appropriate and vessel-specific. As such, future field studies should be dedicated to those under-represented species (like most porpoise and all beaked whale species) and vessel types (such as fishing, sailing or seismic vessels), making sure to include, where possible, clearer details about the vessels that are being considered, like information on engine size, overall length, speed and hull type. Efforts should also focus on Asian, African, South American, international and polar waters, where evidence is still lacking. With the increase in maritime activities and the expansion to newer, previously inaccessible parts of the ocean, it is also important to extend research efforts to offshore areas and remote regions like the Arctic and Antarctic, where populations have been less subjected (and therefore more sensitive) to anthropogenic pressures [41,280,281]. While these regions are inherently more challenging to survey, coordinated international effort should employ recent technological innovations (such as PAM, high-resolution satellite imagery, or unoccupied aerial systems) to expand data coverage to now provide the opportunity to capture evidence even in such remote areas and cryptic species [243,282]. These technological advances also provide opportunities for continuous, non-invasive, long-term monitoring data that was previously unattainable, for cetaceans and vessels alike. |
| **22) • policies.** | *Thank you for the suggestions. We have now added text to expand on this topic (see lines* ***762-771****):*  Lastly, while this systematic map cannot result in direct suggestions on managing vessel traffic, it provides an established, user-friendly evidence base that is easier to interrogate, as opposed to reading each singular document identified in this review. As such, policy makers and managers, both at the local and international level, now have the means to explore the collated resources and form the basis for developing evidence-based guidance and regulations regarding the impacts on vessels. The outcomes of this systematic map can also support international mitigation efforts that emphasise data acquisition and analysis, potentially securing targeted funding toward understudied species, regions, or vessel types. Additionally, it highlights the need for harmonised reporting, which could promote international coordination and compliance and the formulation of industry bodies' best-practice guidelines. |
| **23) Finally, the manuscript sometimes reads more like a descriptive catalogue than a fully analytical synthesis, in particular the discussion. These issues substantially weaken the scientific robustness and global relevance of the paper. Major revisions are therefore required before the manuscript can be considered for publication.** | *Thank you for the extensive and constructive feedback. We hope that the changes made to address the other comments can now better support the robustness of the analysis and showcase deeper understanding of the issues.* |
| **24) The manuscript claims to focus on “direct impacts from maritime vessels themselves” but the boundary between “direct” and “secondary” impacts is not always clear. For example, noise from vessels is included but noise from vessel-associated activities (e.g., pile driving) is excluded. This distinction can be justified and the rationale needs to be more explicit.** | A further clarification is now given in the “Materials and Methods” when defining eligibility criteria (see lines **168-173**):  - Intervention/Exposure: immediate, mechanistic impacts that result from the physical presence, movement, or operation of a vessel without an intermediary process (direct impacts; e.g. ship strike, vessel noise, disturbance). Impacts arising as a consequence of vessel activity, but not from the vessel's immediate action (indirect/secondary impacts; e.g. air guns, fishing gear) as well as those pertaining to subsistence or commercial whaling were not considered eligible. |
| **25) The manuscript completely omits the impact of sailing boat competitions (e.g. Vendée Globe, The Ocean Race), despite their global footprint, the known collisions and operation at high speeds in ecologically sensitive offshore regions. There are multiple mentions of collisions (sometimes with videos where we can identify the species). Data on these events and vessel trajectories do exist and their exclusion leaves an important gap in the assessment of vessel-related pressures. At minimum, this limitation should be acknowledged and justified.** | *We have now added the following text (highlighted in blue) to address sailboat records specifically (see lines* ***575-579****) and also more generally about valuable data sources that may have been missed (see lines* ***757-761****):*  Similarly, there is also limited available evidence regarding the potential impacts of motorized sailing boats (only one document, [236]). While this could be attributed to the fact that they operate mostly in coastal areas, many cetaceans (across most, if not all, families) also inhabit the same waters, making these vessels a potential source of impact. Instead, the number may be explained by differences in classification: as many sailing vessels use engine propulsion for large portions of their operational time [237], they may have been reported as “motorboats” in some of the analysed documents. It may also be possible that majority of the reports of the impacts of sailing vessels may exist in other forms of grey literature (e.g. blog posts, videos) that were not included in this systematic map. This type of evidence can be imperfect and is generally not included in scientific reviews. However it is still valuable and should be considered when it makes up the majority (if not all) of the available information.  In addition to systematic literature reviews, future studies focusing on data-deficient species or vessel types should also attempt to collate information for any types of documentation and media (including generally disregarded sources like blog posts or videos) as well as conducting interviews with marine users, like has been done to document entanglement and bycatch for example [284-288]. |
|  |  |

**Reviewer #2:**

| **Reviewer’s Comment** | *Our Response* |
| --- | --- |
| **The research and referencing work is impressive. The classification methodology is equally impressive.**  **There is some confusion between the title of the article: Understanding the global threats to cetaceans from maritime vessels: a systematic map, and the discussion within it.**  **Indeed, when reading the title, one expects a compilation of impacts over time, by species or groups of species, type of vessel, etc.** | *Thank you, we wish to avoid any confusion for potential readers and therefore propose changing the title to: “Exploring the evidence of direct threats to cetaceans from maritime vessels: a systematic map”, which we feel is more reflective of the content presented in the study.* |
|  |  |
| **The discussion presents the number of references for each, but no summary of existing data.**  **The title led me to expect tables and maps showing impacts, target species, sources, and their evolution over time. In the end, it is a library summary rather than a scientific review. I would expect a review to summarize existing data and conclusions, not just list available publications.** | *Indeed, this manuscript aims to summarize the distribution of the available evidence and highlight knowledge gaps in the research on vessel impacts on cetaceans. As a systematic map, the scope of the methodology is to quantify available evidence rather than evaluating such evidence and summarising its results. The authors recognise that the distinction would benefit from further clarity and as such we have added some text to the Introduction (highlighted in blue) to clarify why a systematic map is more suitable in this case (see lines* ***142-146****) and to the Discussion to highlight how this manuscript can help guide future systematic literature reviews (in which a full meta-analysis can be carried out) (see lines* ***734-761****):*  Previous reviews have focussed on describing the effects of specific type of impact [144] or quantifying evidence available for a particular species or group of recipient species [12], vessel type or activity [12,32], or defined geographic areas [145]. Few, however, have taken a holistic approach, assessing global literature on marine vessel impacts across all vessel types and cetacean species. This study aims to, for the first time, synthesise the currently available evidence for all marine cetaceans, all vessel types and all geographies, allowing for a fuller understanding of this issue. Given the large and highly varied body of literature on this topic, a systematic map was used, over a more extensive literature review, in order to better highlight knowledge gaps and quantify the spatial distribution of the evidence.  In addition to guiding new empirical research, this systematic map can also guide future systematic evidence analysis. Firstly, future synthesis should account for different responses from the animals, with clear distinctions between behavioural, social or physiological responses, vocalization changes, and lethal or sublethal injuries, as well as short- or long-term consequences. Secondly, future reviews should explore seasonal patterns in the available evidence and the possible lack of data during months where survey conditions are commonly unfavourable (like boreal winter, austral summer, monsoon seasons). High-resolution spatial patterns should also be investigated to assess possible biases at a local scale and between inshore and offshore waters, which necessitates recording the full extent of the study area rather than a central location. Thirdly, with the emergence of new technologies, future synthesis could include information of the data sources used for both cetaceans (e.g. land- or ship-based studies, visual or acoustic, eDNA) and vessels (e.g. land- or boat-based surveys, AIS data), with particular interest on temporal and spatial patterns. It will also be important to compare distribution of empirical and predictive evidence, as that is likely to differ between vessel types, species (or species groups) and response types. For example, research using AIS data might be more predictive in nature, and studies on elusive species might rely more strongly on modelling due to the lack of observational records. At a smaller scale, future systematic literature reviews should focus on assessing potential knowledge gaps at the population and sub-population level for those apparently better-studied species, like humpback whales, bottlenose dolphins, killer whales and sperm whales. For this to happen, a higher spatial resolution than the one used in the current study is needed. In particular, extracting information of the size on the study area (rather than the centre location) would allow to better evaluate overlap with both high vessel traffic zones and protected areas, to assess whether cetaceans are being studied and protected where they are actually more vulnerable. In addition to systematic literature reviews, future studies focusing on data-deficient species or vessel types should also attempt to collate information for any types of documentation and media (including generally disregarded sources like blog posts or videos) as well as conducting interviews with marine users, like has been done to document entanglement and bycatch for example [284–288]. |
| **l.65 : Precise the environmental impacts. As the authors go on to detail for cetaceans, just mention the impacts here.** | *Added a brief list of other environmental impacts not related to cetaceans (now lines* ***66-69****):*  Vessel activity can result in notable environmental impacts, such as increased noise, chemical and air pollution, introduction of non-native species, disturbance to seabeds and benthic communities, collision with marine megafauna (including penguins, turtles, pinnipeds, sharks, sirenians and cetaceans) [30-32]. |
| **l.73: Precise, in few words, the negative impact on cetaceans.** | *A brief list of potential impacts has been added, thank you for the suggestion (now lines* ***75-78****):*  A broad range of threats have been connected to maritime traffic, and subsequently documented for many marine species but cetaceans as a group have been identified as being particularly vulnerable to direct threats from the vessels themselves such as: underwater noise [13,35–44], discharges and contaminants [17,45,46], and vessel strikes [14–16,18,20–22,47–55]. In addition, there is a large body of evidence showing that activities associated with different vessel types such as pile driving, seismic surveys, sonar, dredging and fishing gear can also have significant negative and diverse impacts on cetaceans (e.g. displacements from core habitats, changes in vocalization, acoustic masking and entanglement [35, 56-64]). |
| **l.95: I will place the quote [66] after this sequence: “ranges as the vessel”** | *This has been moved (now lines* ***105****).* |
| **l.125: please put a comma after “by vessels”.** | *This has been added (now lines* ***134****).* |
| **l.208: Why choose to group Physeteridae and Kogiidae together when their habitats are different and the latest publications on classification suggest differentiating between them?** | *They were initially grouped together due to their deep-diving behaviour, but on reflection we now agree that these two groups should not have been pooled together. Species are now separated by family to ensure consistency and comparability.* |
| **Table 1: Have you made a distinction between traditional hulls and those using foils when it comes to sailing boats? Are there any usable studies?** | *This would be really interesting to consider, however, sailing boat hull type was rarely mentioned (nor for many of the vessel types), with the only exception being hydrofoils. However we do agree that this would be a very interesting aspect to research on and added it as a suggestion for future studies (see lines* ***699-703****).*  Different cetacean species exhibit varied sensitivities to vessel disturbances [256,276,277], and vessel types differ substantially in their size, speed, noise frequency, and operational patterns, which may result in different impacts [278,279]. This heterogeneity necessitates conservation approaches that are nuanced and adaptive, ensuring that mitigation measures are both species-appropriate and vessel-specific. As such, future field studies should be dedicated to those under-represented species (like most porpoise and all beaked whale species) and vessel types (such as fishing, sailing or seismic vessels), making sure to include, where possible, clearer details about the vessels that are being considered, like information on engine size, overall length, speed and hull type. |
| **l.419: please indicate the proportion. This will help the reader.** | *Thank you for the suggestion, this has been added (now line* ***441****).* |
| **l.452: The use of new techniques (acoustics, eDNA) has also made it possible to study certain species. These techniques have emerged recently, or have been in use for less time than traditional monitoring methods involving direct observation. It would have been interesting to include the techniques used in the results, or even the publication dates associated with the techniques. The same applies to the chronology of the number of studies by species or groups of species.** | *We agree this would be really interesting to look at, indeed there is so much information contained within these papers that we would love to explore further but they were beyond the scope and capacity of the current study but now that the literature has been collated with some updates it could something we would seek to consider for future work, and perhaps moving beyond a systematic map into a full review would enable us to take a deeper dive of the information contained within this sizeable body of literature. We have also acknowledged this as a suggestion for future systematic maps and literature reviews (text highlighted in blue; see lines* ***743-746****):*  In addition to guiding new empirical research, this systematic map can also guide future systematic evidence analysis. Firstly, future synthesis should account for different responses from the animals, with clear distinctions between behavioural, social or physiological responses, vocalization changes, and lethal or sublethal injuries, as well as short- or long-term consequences. Secondly, future reviews should explore seasonal patterns in the available evidence and the possible lack of data during months where survey conditions are commonly unfavourable (like boreal winter, austral summer, monsoon seasons). High-resolution spatial patterns should also be investigated to assess possible biases at a local scale and between inshore and offshore waters, which necessitates recording the full extent of the study area rather than a central location. Thirdly, with the emergence of new technologies, future synthesis could include information of the data sources used for both cetaceans (e.g. land- or ship-based studies, visual or acoustic, eDNA) and vessels (e.g. land- or boat-based surveys, AIS data), with particular interest on temporal and spatial patterns. It will also be important to compare distribution of empirical and predictive evidence, as that is likely to differ between vessel types, species (or species groups) and response types. For example, research using AIS data might be more predictive in nature, and studies on elusive species might rely more strongly on modelling due to the lack of observational records. |
| **L.496-497: But is there a direct impact from fishing boats? It would be interesting to clarify this here.** | *Indeed, some studies have looked at possible collision events, or disturbance due to the presence of fishing boats returning to the harbour. We have added some examples now (see lines* ***563-568****):*  For other vessel types, the relatively fewer records may reflect the fact that the focus of the study has been on the activity being performed, rather than on interactions between cetaceans and the vessels themselves. For instance, while fishing gear and of entanglement are widely recognised as main threats to cetaceans and much effort has been put into understanding and mitigating their impacts [230–232], this review identified fishing boats as some of the least represented vessel types. This however does not automatically translate in a lower risk associated with fishing boats, as the available records provide evidence of collisions with cetaceans as well as behavioural responses and changes in vocalizations [230,237-239] |
